# Herbicide resistance and biodiversity: agronomic and environmental aspects of genetically modified herbicide-resistant plants

**DOI:** 10.1186/s12302-016-0100-y

**Published:** 2017-01-21

**Authors:** Gesine Schütte, Michael Eckerstorfer, Valentina Rastelli, Wolfram Reichenbecher, Sara Restrepo-Vassalli, Marja Ruohonen-Lehto, Anne-Gabrielle Wuest Saucy, Martha Mertens

**Affiliations:** 1 FSP BIOGUM Universität Hamburg, Ohnhorststr. 18, 22609 Hamburg, Germany; 2 Umweltbundesamt GmbH/Environment Agency Austria (EAA), Spittelauer Lände 5, 1090 Vienna, Austria; 3 Italian National Institute for Environmental Protection and Research (ISPRA), Via Vitaliano Brancati 48, 00144 Rome, Italy; 4 Federal Agency for Nature Conservation (BfN), Konstantinstrasse 110, 53179 Bonn, Germany; 5 3130 1/2 Roberts Ave, Culver City, 90232 USA; 6 Natural Environment Centre, Finnish Environment Institute (SYKE), PO Box 140, FI-00251 Helsinki, Finland; 7 Federal Office for the Environment (FOEN), 3003 Bern, Switzerland; 8 Institut für Biodiversität–Netzwerk e.V. (ibn), Nußbergerstr. 6a, 93059 Regensburg, Germany

**Keywords:** Herbicide-resistant crops, Herbicide resistance, Genetically modified crops, Glyphosate, Biodiversity, Farmland biodiversity, Agriculture, Agricultural practice, Sustainability, Pollination

## Abstract

Farmland biodiversity is an important characteristic when assessing sustainability of agricultural practices and is of major international concern. Scientific data indicate that agricultural intensification and pesticide use are among the main drivers of biodiversity loss. The analysed data and experiences do not support statements that herbicide-resistant crops provide consistently better yields than conventional crops or reduce herbicide amounts. They rather show that the adoption of herbicide-resistant crops impacts agronomy, agricultural practice, and weed management and contributes to biodiversity loss in several ways: (i) many studies show that glyphosate-based herbicides, which were commonly regarded as less harmful, are toxic to a range of aquatic organisms and adversely affect the soil and intestinal microflora and plant disease resistance; the increased use of 2,4-D or dicamba, linked to new herbicide-resistant crops, causes special concerns. (ii) The adoption of herbicide-resistant crops has reduced crop rotation and favoured weed management that is solely based on the use of herbicides. (iii) Continuous herbicide resistance cropping and the intensive use of glyphosate over the last 20 years have led to the appearance of at least 34 glyphosate-resistant weed species worldwide. Although recommended for many years, farmers did not counter resistance development in weeds by integrated weed management, but continued to rely on herbicides as sole measure. Despite occurrence of widespread resistance in weeds to other herbicides, industry rather develops transgenic crops with additional herbicide resistance genes. (iv) Agricultural management based on broad-spectrum herbicides as in herbicide-resistant crops further decreases diversity and abundance of wild plants and impacts arthropod fauna and other farmland animals. Taken together, adverse impacts of herbicide-resistant crops on biodiversity, when widely adopted, should be expected and are indeed very hard to avoid. For that reason, and in order to comply with international agreements to protect and enhance biodiversity, agriculture needs to focus on practices that are more environmentally friendly, including an overall reduction in pesticide use. (Pesticides are used for agricultural as well non-agricultural purposes. Most commonly they are used as plant protection products and regarded as a synonym for it and so also in this text.)

**Electronic supplementary material:**

The online version of this article (doi:10.1186/s12302-016-0100-y) contains supplementary material, which is available to authorized users.

## Preliminary remark

Together with the supplement, the present paper is a summary and an update of a comprehensive technical report which was previously published by the German Federal Agency for Nature Conservation BfN, the Austrian Environment Agency EAA, and the Swiss Federal Office for the Environment FOEN [1]. Based on this technical report (see Additional file 1), some members of the Interest Group GMO within the EPA and ENCA networks,^1^ drafted a position paper which highlights key messages regarding the environmental impacts of the cultivation of genetically modified herbicide-resistant plants [2, 3]. Acting upon the key messages should improve the current environmental risk assessment of these plants. The position paper was recently addressed to relevant EU bodies with the aim to ensure adequate protection of the environment in the future.

Most of the members of the IG GMO within the EPA and ENCA networks are involved in the risk assessment of GMOs in the EU and other European countries. Hence, the group consists of agencies responsible for the authorization of GMO releases as well as public institutions that provide scientific support to national administrations, e.g. as regards risk assessment.

This paper summarizes the lessons learned from the experience with the use of GM plants resistant to the herbicides glyphosate and glufosinate. It is based on a more detailed paper that can be accessed as a supplement to this article. Ongoing discussions about the food and feed safety of GM crops and the concept of substantial equivalence are not in the realm of this paper.

Throughout this document, the terms “herbicide resistance” and “herbicide tolerance” are used as defined by the Weed Science Society of America [4]; both terms are not used synonymously with respect to a particular response to a herbicide; they rather distinguish naturally occurring “tolerance” from engineered “resistance”.

## Review

### Agreements and regulations covering biodiversity protection

Conservation of biodiversity is high on the agenda of international and national environmental policies though not very present in public awareness. The need to protect biodiversity and stop the loss was acknowledged in the Convention on Biological Diversity (CBD), internationally agreed on in 1992, and underscored by relevant decisions since then^2^ (the Convention entered into force in 1993). The Cartagena Protocol on Biosafety (CPB), adopted by the Parties to the CBD in 2000 and entering into force in 2003, seeks to protect biological diversity from potential risks posed by living modified organisms (LMOs), specially focusing on transboundary movement. Moreover, the CPB aims to facilitate information exchange on LMOs and procedures to ensure that countries can make informed decisions before they agree to import LMOs. Actually, 195 nations plus the EU are Parties to the CBD and 169 plus the EU to the Cartagena Protocol.

In the EU, the deliberate release into the environment of genetically modified organisms (GMOs) is regulated by the Directive 2001/18/EC and the Directive (EU) 2015/412. Referring to the precautionary principle, the Directive 2001/18/EC aims at the protection of human and animal health and the environment. In the course of the environmental risk assessment, intended and unintended as well as cumulative long-term effects relevant to the release and the placing on the market of GMOs have to be considered comprehensively.

Most commercially planted genetically modified (GM) crops are either herbicide-resistant (HR) or insect-resistant (IR), many carrying both traits. Based on recent data and experience, there are concerns that HR crops promote the further intensification of farming and may therefore increase pressure on biodiversity.

### Herbicide-resistant crops

Herbicide resistance is the predominant trait of cultivated GM crops and will remain so in the near future. GM crops resistant to the broad-spectrum herbicides glyphosate and glufosinate have first been cultivated commercially in the 1990s [5], and GM crops with resistance to other herbicides are under development [6], or already on the market, with various HR traits increasingly combined in one crop [7]. Another, more recent strategy is the development of plants that are resistant to high concentrations of glyphosate without exhibiting a yield drag [8, 9].

Glyphosate inhibits 5-enolpyruvylshikimate-3-phosphate synthase (EPSPS), an enzyme of the shikimate pathway for biosynthesis of aromatic amino acids and phenolics in plants and microorganisms. This enzyme is not present in human or animal cells [10]. Glufosinate ammonium is an equimolar, racemic mixture of the d- and l-isomers of phosphinothricin (PPT). The l-isomer inhibits plant glutamine synthetase, leading to the accumulation of lethal levels of ammonia [11].

To confer resistance to glyphosate, most glyphosate-resistant crops express a glyphosate-insensitive EPSPS derived from *Agrobacterium* spp., some also the glyphosate-degrading enzyme glyphosate oxidoreductase (GOX) and/or the enzyme glyphosate acetyltransferase (GAT) that modifies glyphosate. In addition, various crops have also been transformed with one of the two bacterial genes *pat* or *bar* from *Streptomyces* spp. conferring resistance to glufosinate-based herbicides. These genes encode the enzyme phosphinothricin acetyl transferase (PAT) which detoxifies l-PPT. Other transgenes contained in HR crops confer resistance to ALS inhibitors^3^ (*gm*-*hra* gene), 2,4-D^4^ (*aad*-1 and *aad*-12 genes) or to dicamba (*dmo* gene).

While many transgenic HR crop species have been tested in the field, only four are widely grown commercially since the late 1990s: soybean, maize, cotton, and canola [12]. In 2013, of the 175.2 million ha global GM crop area, about 57% (99.4 million ha) were planted with HR varieties and another 27% (47 million ha) with stacked HR/IR crops [13]. Hence, 84% of the GM crops carried HR genes (146.4 million ha). HR soybean is the dominant GM crop and grown mainly in North and South America, making up about 80% of the global soybean area and 46% of the total GM crop area [12]. In GM maize and GM cotton, HR traits are often combined with IR genes. In the US, HR crops such as alfalfa, sugar beet, creeping bentgrass, and rice, are already deregulated and on the market or pending for deregulation [7].

### Yields of HR crops

Contrary to widespread assumptions, HR crops do not provide consistently better yields than conventional crops. Increased yield is not the main reason for farmers to adopt HR crops. If there are yield differences between HR and conventional crops, they may be due to various factors, such as scale and region of growing, site and size of farms, soil, climate, tillage system, weed abundance, genetic background/varieties, crop management, weed control practice, farmer skills, and the education of the farm operators. Reviewing data about the agronomic performance of GM crops, Areal et al. [14] concluded that although GM crops, in general, perform better than conventional counterparts in agronomic and economic (gross margin) terms, results on the yield performance of HR crops vary. A consistent yield advantage for HR crops over conventional systems could not be demonstrated [15–17].

The actual yield reduction in RoundupReady soybean observed in some studies [15] might be due to several causes: (i) the present resistance gene in the first generation of RoundupReady line (40-3-2) [18] and (ii) reduced nodular nitrogen fixation upon glyphosate application [19] and/or (iii) a weaker defence response [20]. Application of glyphosate seemed to affect nodule number and mass which have been correlated with nitrogen fixation [21] and cause the symptom of “yellow flashing” which leads to a decrease in grain yield (see discussion in [9]). The second generation RR2Y soybean (MON 89788) was introduced to provide better yields, but when tested in the greenhouse, different cultivars of RR2Y performed less well than RR 40-3-2 [22].

### Eco-toxicological attributes of complementary herbicides

Impacts of HR crops on biodiversity are possible through the altered herbicide management option, that is, application of a broad-spectrum herbicide during crop growth and its impacts on weed abundance and diversity. These impacts, also called indirect effects, are dealt with later in this text. Direct impacts relate to the toxicity of the herbicide, of residues, and breakdown products. First, an update of eco-toxicological attributes and direct effects of relevant complementary herbicides of HR crops is given.

#### Glyphosate

Glyphosate (C_3_H_8_NO_5_P; *N*-(phosphonomethyl) glycine), a polar, water soluble organic acid, is a potent chelator that easily binds divalent cations (e.g. Ca, Mg, Mn, and Fe) and forms stable complexes [23]. In addition to the active ingredient (a.i.) that can be present in various concentrations, herbicides usually contain adjuvants or surfactants that facilitate penetration of the active ingredient through the waxy surfaces of the treated plants. The best known glyphosate containing herbicides, the Roundup product line, often contain as a surfactant polyethoxylated tallow amine (POEA), a complex mixture of di-ethoxylates of tallow amines characterized by their oxide/tallow amine ratio, that is significantly more toxic than glyphosate [24]. The toxicity of formulations to human cells varies considerably, depending on the concentration (and homologue) of POEA [25]. Data from toxicity studies performed with glyphosate alone and over short periods of time may thus conceal adverse effects of the herbicides. Glyphosate degradation is reported to be rapid (half-lives up to 130 days) [3], but its main metabolite aminomethylphosphonic acid (AMPA) degrades more slowly. Both substances are frequently and widely found in US soils, surface water, groundwater, and precipitation [26]. Recently, the widespread occurrence of POEA and the persistence of POEA homologues in US agricultural soils have been reported [27] with currently unknown and unexplored consequences.

Inhibition of the enzyme EPSPS and disruption of the shikimate pathway impacts protein synthesis and production of phenolics, including defence molecules, lignin derivatives, and salicylic acid [28]. Glyphosate impacts plant uptake and transport of micronutrients (e.g. Mn, Fe, Cu, and Zn) whose undersupply can reduce disease resistance and plant growth [20, 23]. In Argentine soils, residue levels of up to 1500 µg/kg (1.5 ppm) glyphosate and 2250 µg/kg (2.25 ppm) AMPA have been found [29].

Glyphosate affects the composition of the microflora in soil and gastrointestinal tracts differently, suppressing some microorganisms and favouring others [30, 31]. This is likely linked to varying sensitivities of bacterial EPSPS enzymes to glyphosate [32]. In the RoundupReady soybean system, the bacterial-dependent nitrogen fixation and/or assimilation can be reduced [33]. Impacts of glyphosate on fungi vary also, depending on study sites, species, pathogen inoculum, timing of herbicide application, soil properties, and tillage [28]. Mycorrhizal fungi seem to be sensitive to glyphosate [34], while others, including pathogenic *Fusarium* fungi, may be favoured under certain conditions since glyphosate may serve as nutrient and energy source [30]. The microbial community of the gastrointestinal tract of animals and humans may be severely affected, if, as reported by Shehata et al. for poultry microbiota in vitro [31], pathogenic bacteria (e.g. Salmonella and Clostridium) are less sensitive to glyphosate than beneficial bacteria, e.g. lactic acid bacteria. For this reason, studies on glyphosate effects on the gut microbiome of other species are needed.

Glyphosate-based herbicides can affect aquatic microorganisms both negatively (e.g. total phytoplankton and nitrifying community) and positively (e.g. cyanobacteria) [35, 36], with surfactants such as POEA being significantly more toxic than the active ingredient itself [37]. In studies where *Daphnia magna* were fed glyphosate residues for the whole life-cycle, the parameters growth, reproductive maturity, and offspring number were impaired [38]. Amphibians are particularly at risk, since shallow temporary ponds are areas where pollutants can accumulate without substantial dilution. Sublethal concentrations of glyphosate herbicides can cause teratogenic effects and developmental failures in amphibians and impact both larval and adult stages [39]. Environmentally relevant levels of exposure to both glyphosate and Roundup have led to major changes in the liver transcriptome of brown trout, reflective of oxidative stress, and cellular stress response [40]. Simultaneous exposure to glyphosate-based herbicides and other stressors can induce/increase adverse impacts on fish [41] and amphibians [42].

Glyphosate application reduced the number and mass of casts and reproductive success of earthworm species that inhabit agroecosystems [43]. Impacts on arthropods, among them beneficial land predators and parasites, vary [44]. Exposure to sublethal glyphosate doses impairs behaviour and cognitive capacities of honey bees [45]. Acute toxicity of glyphosate to mammals is lower relative to other herbicides. In recent years, however, glyphosate-based herbicides have been reported to be toxic to human and rat cells, impact chromosomes and organelle membranes, act as endocrine disruptors, and lead to significant changes in the transcriptome of rat liver and kidney cells [25, 46, 47]. Negative effects of glyphosate on embryonic development after injection into *Xenopus laevis* and chicken embryos have been linked to interference of glyphosate with retinoic acid signalling that plays an important role in gene regulation during early vertebrate development, also showing that damage can occur at very low levels of exposure [48]. The International Agency for Research on Cancer (IARC) concluded in a recent report that glyphosate is probably carcinogenic to humans [49]. When mandated by the European Commission to consider IARCS’s conclusion, EFSA identified some data gaps, but argued that, based on its own calculations about glyphosate doses humans may be exposed to, glyphosate is unlikely to pose a carcinogenic hazard to humans [50]. The current concerns over the use of glyphosate-based herbicides are summarized in a recent paper [51], which concludes that glyphosate-based herbicides should be prioritized for further toxicological evaluation and for biomonitoring studies.

#### Glufosinate ammonium

l-PPT glufosinate inhibits glutamine synthetase of susceptible plants and results in accumulation of lethal levels of ammonia [11]. Less data on eco-toxicity of glufosinate is available compared to glyphosate, presumably due to the significantly lower use of glufosinate. The formulated product is known to be (slightly) toxic to fish and aquatic invertebrates. Glufosinate has been shown to suppress some soil microorganisms, whereas others exhibited tolerance [52]. Some fungal pathogens seem to be reduced by glufosinate, potentially due to inhibition of glutamine synthetase, similar to the inhibition in plants [53]. Glufosinate may impact predatory insects, mites, and butterflies [54, 55].

Glufosinate ammonium has the potential to induce severe reproductive and developmental toxicity in rats and rabbits [56]. Because of its reproductive toxicity, use of glufosinate will be phased out in the EU by September 2017 [57]. In other countries, however, glufosinate use may not be discontinued as glufosinate-resistant crops are increasingly grown in reaction to the ever greater number of glyphosate-resistant weeds [7, 58].

#### Other herbicides

The increasing use of “old” herbicides such as synthetic auxins, expected in the course of US deregulation of crops resistant to 2,4-D or dicamba, raises serious concerns. Synthetic analogues of the plant hormone auxin cause uncontrolled and disorganized plant growth finally killing sensitive plants, e.g. broadleaf weeds. The herbicide 2,4-D is 75 times and dicamba 400 times more toxic to broadleaf plants than glyphosate [59]. Both herbicides are highly volatile, thus increasing the potential for damage to non-target organisms due to spray drift. Sensitive crops, vegetables, ornamentals, and plants in home gardens could be damaged and both plant and arthropod communities in field edges and semi-natural habitats affected [60]. Whether a new formulation with lower volatility to be used in resistant crops, e.g. Enlist Duo comprising 2,4-D and glyphosate, and special stewardship guidelines will help reduce adverse herbicide effects, is highly questionable [59] since lower volatility of a substance may reduce exposure, but not toxicity, and stewardship programs address resistance issues in the target organisms and not toxicity issues.

The herbicides 2,4-D and 2,4,5-T (2,4,5-trichlorophenoxyacetic acid) each accounted for about 50% of Agent Orange, the herbicide product sprayed by the US military in the jungle in Vietnam. Agent Orange contained highly toxic impurities, including dioxins and furans. Such impurities in actual 2,4-D containing herbicides are still a concern, especially in herbicides manufactured outside the EU and US [61]. Recently, IARC [62] classified 2,4-D as a “possible human carcinogen,” a classification which is not shared by EFSA [63]. Due to potential synergistic effects between the two ingredients in Enlist Duo on non-target plants, the US Environmental Protection Agency has considered taking legal action to revoke the registration of this herbicide mix [64].

### Impacts on agricultural practice and agronomy

HR crops can have various impacts on the agricultural practice and agronomy, including weed control, soil tillage, planting, crop rotation, yield, and net income. These interdependent factors influence to which degree and under which circumstances HR crops are adopted and should be taken into account, when impacts of HR crops on biodiversity are considered comprehensively.

Resistance to the broad-spectrum herbicides glyphosate and glufosinate allows previously sensitive crops to survive their application, facilitating weed control and giving the farmer more flexibility, e.g. by extending the time window for spraying and post-emergence application. Conservation tillage, often recommended to reduce soil erosion and to save costs and energy, has increased and might even further expand if more HR crops are grown, as they are well adapted to low tillage systems. From 1996 to 2008, adoption of conservation tillage in US soybean cultivation increased significantly [58].

In the US, the most often stated reasons for the adoption of HR crops were improved and simplified weed control, less labour and fuel cost, no-till planting/planting flexibility, yield increase, extended time window for spraying, and in some cases decreased pesticide input [65]. Labour reduction may allow generating off-farm income [66]. In the beginning, weed resistance management did not seem that important to farmers, although weeds had become resistant to commonly used selective herbicides before [6]. Farmers were likely guided by the industry’s argument that, for a couple of reasons, among them glyphosate’s unique properties, glyphosate-resistant weeds would not evolve, at least not very rapidly [67]. Reasons for adoption of HR crops in South America were similar to those mentioned above [68]. Moreover, lack of patent protection of GM seeds facilitated the introduction of HR soybean in Argentina, as seeds could be saved for planting and resale, and could also enter the black market from where they were smuggled into Brazil [69].

Crop rotation helps maintain high productivity by reducing pesticide use and fertilizer input and can reduce pest and pathogen incidences, weed infestation, and selection pressure for weed resistance to herbicides [58]. However, in regions where HR crops are widely adopted, there is a clear trend toward monoculture and crop rotation and diversification are reduced [59]. In the US, in very large areas, crop rotation comprises only glyphosate-resistant crops, the most common rotation being HR soybean to HR corn [66]. In Argentina, within a few years, continuous HR soybean replaced 4.6 million ha of land initially dedicated to other crops, leading to a noticeable homogenization of production and landscapes [68].

### Weed control patterns and herbicide use

HR crops are advertised as being environmentally friendly due to less herbicide use, compared to conventional crops. However, actual trends rather support the opposite. Changes in overall amount of herbicides used are difficult to assess since different herbicides are applied at different rates. Nevertheless, reports show that with the introduction of HR crops in the US in 1996, lower amounts of herbicides were applied during the first years, with glyphosate replacing other herbicides [70]. However, since then, overall herbicide use in HR crops has increased: From 1998 to 2013, the increase in amounts (kg/ha) of active ingredient (a.i.) in HR soybean was 64%, compared to 19% in conventional soybean [71]. The cultivation of HR soybean, maize, and cotton led to an increased herbicide use in the US by an estimated 239 million kg in 1996–2011, compared to non-HR crops, with HR soybean accounting for 70% of the total increase [72].

Global glyphosate use increased too. While from 1995 to 2014, US agricultural use of glyphosate rose ninefold to 113.4 million kg, global agricultural use rose almost 15-fold to 747 million kg, with more than 50% accounted for by use on HR crops [73]. In Argentina, glyphosate use more than doubled from 2000 to 2011, due to the steady increase of the cultivation area of RoundupReady soybeans [74]. In case HR crops would be grown in Europe, it is estimated that herbicide use would rise significantly. If HR crop introduction were accompanied by resistance management, herbicide use would rise by 25%, and if it were unlimited as in the US, the increase would be 72% [75].

In addition, increased weed resistance to glyphosate leads to changes in the mix, total amount, cost, and overall environmental profile of herbicides applied to HR crops [6, 71]. In 2013, almost two-thirds of RoundupReady soybean crops received an additional herbicide treatment, compared to 14% in 2006 [71], e.g. the use of 2,4-D increased from 2002 to 2011 by almost 40% [58]. With the introduction of additional HR traits, “old” herbicides such as 2,4-D, dicamba, ACCase,^5^ and ALS inhibitors are used more frequently again. After deregulation in the USA of 2,4-D-resistant GM soybean and corn, 2,4-D amounts applied in the US could triple by 2020 compared to 2011, with glyphosate use remaining stable [58]. Use of 2,4-D on corn could increase over 30-fold from 2010 levels [72].

### Changes in weed susceptibility

Both non-selective herbicides glyphosate and glufosinate are effective on a wide range of annual grass and broadleaf weed species. The simplicity and effectiveness of weed control in HR cropping systems can be undermined in several ways: (i) by shifts in weed communities and populations resulting from the selection pressure by the applied herbicides, (ii) by escape and proliferation of transgenic plants as weedy volunteers, and (iii) by hybridization with—and HR-gene introgression into—related weedy species. While point (i) indicates changes in biodiversity, points (ii) and (iii) could increase the overall herbicide use in chemical weed management and thereby affect biodiversity further.

#### Selection of resistance and weed shifts

In general, increased reliance on herbicides for weed control leads to a shift in weed species composition. Less sensitive species and populations survive herbicide sprayings and subsequently grow and spread, whereas more sensitive species disappear. In early 2016, a total of 249 weed species (with 464 biotypes) resistant to various herbicides have been recorded, occupying hundreds of thousands of fields worldwide. Many of these biotypes are resistant to more than one herbicide mode of action [76]. Resistance genes can spread by hybridization between related weed species [77] and possibly accumulate in certain biotypes.

Although glyphosate (and glufosinate) have long been considered to be low-risk herbicides with regard to the evolution of resistance [78], at least 34 glyphosate-resistant weed species (more than 240 populations) have been confirmed today, observed on millions of hectares, and increasingly associated with HR crop cultivation [76]. Many of them express resistance to other herbicide classes, too. In the US, the true area infested likely exceeds 28 million ha [79] by a sizable margin. In particular, glyphosate-resistant palmer amaranth (*Amaranthus palmeri*) creates control problems and poses a major economic threat to US cotton production [58]. Recently, two weed species resistant to glufosinate have been described, among them one population resistant also to glyphosate [76].

The molecular and genetic mechanisms of resistance to glyphosate are very diverse and can co-occur [77, 80]. Mutations in the EPSPS target site [81], increased EPSPS mRNA levels [82], EPSPS gene amplification [83], delayed glyphosate translocation [84], sequestration of glyphosate in vacuoles [85], and degradation in the plant [86] have been described. The increased glyphosate use has also promoted species shift among the weed flora, and several grass and broadleaf weeds are becoming problematic weeds [87].

### Resistance management

In the beginning of HR crop cultivation, resistance management was not considered to be an issue [67, 88], but this has later changed [89, 90]. For more than a decade now, weed scientists are recommending that farmers should implement an integrated weed management approach that consists of “many little hammers”. These “hammers” include crop and herbicide rotation, mechanical weeding, cover crops, intercropping, and mulching [91, 92]. But continuous HR cropping became common in the Americas, and farmers often simply resorted to higher glyphosate doses, additional applications (often both) and combined use of other herbicides [93]. Paraquat and synthetic auxins are recommended in tank mixtures or in rotation with glyphosate, but resistance to these herbicides is about as common as resistance to glyphosate [76]. New herbicides will not be commercialized within the near future, due to the increased development costs and the challenge to find suitable substances that comply with the stricter regulatory standards for weed efficacy and environmental and toxicological safety [6].

In this context, it is noted that companies increasingly develop and commercialize GM crops that resist higher glyphosate doses or that contain stacked HR traits, such as resistance to glyphosate and/or glufosinate, in part combined with resistance to 2,4-D, dicamba, ACCase inhibitors or HPPD^6^ inhibitors [6, 7, 9]. But as resistance to these herbicides is already common [76], stacking of HR traits and increased use of herbicides other than glyphosate will not reduce the selection pressure on weeds or decrease overall herbicide amounts applied. In addition, merely rotating herbicides may exacerbate resistance problems by selecting for broader resistance mechanisms in weeds [94].

Against this background, integrated weed management is strongly recommended and seems to be the only sensible strategy in the long-term. Cropping systems that employ such an approach are competitive with regard to yields and profits to systems that rely chiefly on herbicides [59]. A four-year crop rotation scheme (maize-soybean-small grain + alfalfa–alfalfa) not only helped reduce herbicide applications and fertilizer input, but also provided similar or even better yields and economic output, compared to the two-year maize-soybean rotation common in the US [95]. However, although tools for weed control other than herbicides are clearly needed, use of herbicides is still the main weed management method and the number of papers dealing with chemical control eclipse those on any other method [96].

### Seed escape and proliferation of HR plants

Seed escape and proliferation of HR plants can create severe management problems, especially with persistent crops. Volunteers, that is, crop plants in the field emerging from the previous crop, create problems when the following crop is a different species or a different variety of the same species. Volunteer management will become more complex if both volunteer plants and crops are resistant to the same herbicide. Crops with characteristics such as shattering and seed persistence are particularly likely to emerge as volunteers. Oilseed rape readily produces volunteers and feral plants, due to its high seed production, high seed losses during harvest and transport, and its secondary dormancy [97]. HR oilseed rape plants have been found up to 15 years after experimental releases, despite regular control of the fields for volunteers [98, 99]. The recently reported incidence of oilseed rape seed contamination by the non-approved OXY-235 variety (resistant to oxynil herbicides) in the EU might be traced back to field trials in France in the nineties [100], indicating that volunteers may emerge even after almost 20 years. Seed spill can also occur outside the fields and along transport routes, potentially leading to HR feral plants that may persist over large spatial and temporal scales [101]. HR feral oilseed rape plants have been found along transport routes in the US [102], in Switzerland [103] and Japan [104], in regions where they had never been grown.

### HR-gene flow to volunteers, neighbouring crops or interfertile weeds

Gene flow from HR crops is a special aspect of agrobiodiversity and relevant for the purity of genetic resources. The frequency of outcrossing depends on the crop species in question and its pollination system, the distance to simultaneously flowering volunteers or relatives, and variables such as genotype, abundance and foraging behaviour of pollinators, weather conditions, time of the day, and the size of pollen donor and receiving populations. Novel combinations of transgenic events can be formed in the wild [102]. Reviews on gene flow have focused on the main GM crops [105] or on single crop species such as oilseed rape [106], maize [107], rice [108], sugar beet [109], and soybean [110]. As large pollen sources, such as crop fields, interact on a regional scale, and tend to increase gene flow, isolation distances have to be adjusted to this factor [111].

In centres of crop origin and regions where interfertile weeds, which can hybridize with crops, are present, gene flow from crop to weeds should be taken into account. This is true for oilseed rape (*Brassica napus*) and its close relative field mustard (*Brassica rapa*) in many regions of Europe [106]. Once herbicide resistance genes move into weeds, their frequency within local weed populations will increase under selection pressure by the corresponding herbicide. Hybrids do not need to be particularly fit as long as they are able to backcross with the weedy relative, a capacity which is characteristic for many interspecific hybrids. Even genotypes with a lower fitness may survive if the pollen flow is steady and the pollen source is large [112].

In some European regulation frameworks, e.g. according to the Swiss Biosafety regulations, undesired gene flow in itself is considered an adverse effect.^7^

### Agriculture and biodiversity

Intensive high-input farming is a major force driving biodiversity loss and other environmental impacts beyond the “planetary boundaries” [113, 114]. Drivers are e.g. the low number of cropped species, reduced rotation, limited seed exchange between farms, drainage, and landscape-consolidation, and increased use of pesticides. At the same time, agriculture relies on ecosystem functions and services and on biodiversity, including pollination, biological pest control, maintenance of soil structure and fertility, nutrient cycling, and hydrological services [115].

Weeds are part of the biodiversity of the agroecosystem. Although commonly regarded as pests, they offer considerable benefits to the agroecosystem by supporting a range of organisms such as decomposers, predators, pollinators, and parasitoids. They fulfil certain functions within the agroecosystem which becomes obvious when they are missing, e.g. decreasing the antagonists of pest species can increase pesticide inputs as demonstrated by exclusion experiments [116, 117], and lower numbers of pollinators may reduce yield and quality in crops depending on animal pollination [118]. Within the last decades, the diversity of the “associated agricultural flora” (a neutral expression for weeds) and the seed bank in arable soils have been reduced significantly [119, 120]. If the associated flora and arthropods are decreased in terms of abundance and diversity, this will affect the whole food chain including small mammals and farmland birds, the latter being major targets, and important indicators of agricultural change [121]. Organic farming, however, has a large positive effect on biodiversity with plants benefiting the most among taxonomic groups [122].

### Indirect effects of HR agriculture on biodiversity

As outlined above, broad-spectrum herbicides directly affect various organisms. However, as part of the HR weed management system, they also affect biodiversity as a whole. As glyphosate and glufosinate are effective on more weed species than other currently used herbicides or mechanical weeding and than is necessary for crop protection and productivity, they will increase the level of weed suppression. Therefore, HR crops will likely support monocultures and the excessive control of weeds in agricultural environments. Indications of increased loss of biodiversity have been found in the three years Farm Scale Evaluations (FSE), where the effects of HR cropping systems on abundance and species diversity of wild plants and arthropods were investigated across Britain [123, 124]. In glyphosate-resistant sugar beet and fodder beet and in glufosinate-resistant oilseed rape, the wild plant density, biomass, seed rain, and seed bank were lower by one-third to one-sixth than in the conventional counterparts; also less species emerged, compared to conventional management [125–127]. On the other hand, glufosinate-resistant maize showed more diverse weed species, compared to conventional maize sprayed with atrazine. However, atrazine is highly effective on a broad range of plants and no longer approved in the EU. Herbicide drift to field margins is a concern to nature conservation and biodiversity of agricultural landscapes, as field margins and hedgerows often harbour rare plant species [128]. These habitats too were negatively affected in the FSE trials [129].

In the FSE trials, the abundance of arthropods changed in the same direction as their resources and herbivores, pollinators, and beneficial natural enemies of pests were reduced [130]. The FSE findings are supported by results in a 1-year canola field study in Canada, where wild bee abundance was highest in organic fields, followed by conventional fields and lowest in HR crops [131]. This might also impact vertebrates: If weed abundance and spectra are diminished, birds [132] and migrating adult amphibians [39] may have difficulties finding enough seeds or invertebrates for food. A prominent example of indirect effects of HR crops on biodiversity on a large scale is the monarch butterfly case. Recent US data indicate that, within the last decade and in parallel to the widespread and increased adoption of HR crops, the population size of the migratory monarch butterfly (*Danaus plexippus*) has declined significantly, due, at least in part, to the widespread loss of milkweeds (*Asclepias syriaca*) in the Midwest [133–135]. Milkweed is the main food plant of monarch larvae, and the Midwest is the main breeding ground for monarchs. In case HR maize and HR oilseed rape would be widely grown in Europe, a similar scenario has been predicted for the European butterfly Queen of Spain fritillary (*Issoria lathonia*) [136].

### Aspects of sustainable agriculture

The overreliance of HR cropping systems on chemical weed control discourages the use and retention of existing alternative weed management skills. In addition, HR cropping systems are not compatible with mixed cropping systems [137]. Diversification practices, however, such as cover crops, mixed cropping, intercropping, and agroforestry, help retain soil and soil moisture better than intensive cropping and improve resiliency to climate disasters and thus support the structures of the agroecosystem which provide ecosystem services.

Small multifunctional and ecologically managed farms are more productive than large farms, if total output including energy input/output is considered rather than single-crop yield. However, human labour cannot be fully substituted by mechanization in such farming approaches [138, 139]. Davis et al. [95] showed in a nine-year field study in the US corn belt that more diverse rotations including forage legumes enhanced yields of corn and soybean grain by up to 9% and reduced fertilizer application, energy use, and herbicide input significantly. Weed control and profitability remained the same, whereas labour demand was higher.

As pointed out by the International Assessment of Agricultural Knowledge, Science and Technology for Development [140], agriculture is multifunctional and serves diverse needs. But for many years, agricultural science and development have focused on delivering technologies to increase farm-level productivity rather than integrating externalities such as impacts on biodiversity and the relationship between agriculture and climate change. In view of the current challenges, IAASTD concludes that business as usual is not an option. Rather increased attention needs to be directed toward new and successful existing approaches to maintain and restore soil fertility and to maintain a truly sustainable agricultural production. From the data collected and assessed, HR cropping systems seem to be no option for a sustainable agriculture that focuses also on protection of biodiversity. On the contrary, HR crops rather seem to be part of the problem.

## Conclusions

Intensive high-input farming is known as one of the main drivers of the continuous biodiversity loss in agricultural landscapes. Diversity and abundance of the weed flora provide relevant indicators for farmland biodiversity. While HR cropping facilitates weed control for farmers and makes chemical weed management more flexible, it is accompanied by increased herbicide use and less crop rotation. Toxic effects of the complimentary herbicides on non-target organisms, e.g. soil and aquatic organisms have been shown. Due to the widespread use of glyphosate, at least 34 glyphosate-resistant weed species have evolved worldwide. To counter resistance evolution in weeds, integrated weed management is recommended. But continuous and widespread HR cropping is still very common. The commercial trend is to develop new GM crops with stacked HR traits and GM varieties with increased glyphosate resistance. However, this approach will not reduce the overall herbicide amounts used in agriculture. Control problems can also arise due to HR volunteers or feral plants, e.g. HR oilseed rape. In centres of crop origin and regions where sexually compatible plants occur, transfer of HR genes to wild relatives can be expected. Biodiversity will be affected by HR cropping systems by the very efficient removal of weed plants which in turn leads to a further reduction of flora and fauna diversity and abundance. A prominent example in this respect may be the decline of monarch butterfly populations in the US which has been linked to the massive loss of their food plants upon widespread adoption of HR crops. Since it has been shown that HR systems are not compatible with measures to stop the loss of biodiversity on farmland, a more sustainable model of agriculture is needed, which, according to the present experience, cannot reasonably integrate approaches like HR cropping.

## Additional file

**Additional file 1.** Update of the technical report “Agronomic and environmental aspects of the cultivation of genetically modified herbicide-resistant plants”, BfN-Skripten 362. https://www.bfn.de/fileadmin/MDB/documents/service/skript362.pdf.

## Abbreviations

2,4-D: 2,4-dichlorophenoxyacetic acid
ACCase: acetyl CoA carboxylase
ALS: acetolactate synthase
AMPA: aminomethylphosphonic acid
CBD: Convention on Biological Diversity
CPB: Cartagena Protocol on Biosafety
EPSPS: 5-enolpyruvylshikimate-3-phosphate synthase
FSE: Farm Scale Evaluations
GAT: glyphosate acetyltransferase
GMOs: genetically modified organisms
GM: genetically modified
GOX: glyphosate oxidoreductase
HPPD: hydroxyphenylpyruvate dioxygenase
HR: herbicide-resistant or herbicide resistance
IAASTD: International Assessment of Agricultural Knowledge, Science and Technology for Development
IARC: International Agency for Research on Cancer
IG GMO: Interest Group genetically modified organism
IR: insect-resistant
LMOs: living modified organisms
PAT: phosphinothricin acetyl transferase
POEA: polyethoxylated tallow amine
PPT: phosphinothricin
USDA: United States Department of Agriculture

## Footnotes

1 The European Networks of the Heads of Environment Protection Agencies EPA and European Nature Conservation Agencies ENCA. The subset of the Interest Group GMO consisted of the Environment Agency Austria EAA, the Finnish Environment Institute SYKE, the German Federal Agency for Nature Conservation BfN, the Institute for Environmental Protection and Research ISPRA, and the Swiss Federal Office for the Environment FOEN.

2
http://www.cbd.int.

3 Acetolactate synthase (ALS).

4 2,4-dichlorphenoxyacetic acid.

5 Acetyl CoA carboxylase (ACCase)-inhibitors.

6 Hydroxyphenylpyruvate dioxygenase (HPPD).

7 Swiss Federal Act on Non-Human Gene Technology, Art. 6 lit. 3(e).
