# Supplementary material for: Herbicide resistance and biodiversity: agronomic and environmental aspects of genetically modified herbicide-resistant plants
Source: Environ Sci Eur. 2017 Jan 21;29(1):5. doi: 10.1186/s12302-016-0100-y (PMC5250645; doi:10.1186/s12302-016-0100-y)
Supplement: Supplementary file 1 — Additional file 1. Update of the technical report “Agronomic and environmental aspects of the cultivation of genetically modified herbicide-resistant plants”, BfN-Skripten 362. https://www.bfn.de/fileadmin/MDB/documents/service/skript362.pdf. [file 12302_2016_100_MOESM1_ESM.docx]

**SUPPLEMENT**

**Herbicide resistance and biodiversity: Agronomic and environmental aspects of genetically modified herbicide-resistant plants**

# Summary

Farmland biodiversity is an important characteristic when assessing sustainability of agricultural practices and is of major international concern as shown by the Convention on Biodiversity (CBD) and the various decisions since then. Scientific data indicate that agricultural intensification and pesticide use are among the main drivers of biodiversity loss. Given the actual trends in cultivation of herbicide-resistant (HR) crops, the HR crop system did not increase yields significantly and could not reduce herbicide use. Glyphosate-based herbicides have been shown to be toxic to a range of organisms and to adversely affect soil and intestinal microflora and plant resistance to disease. Glufosinate exhibits reproductive toxicity to mammals and will be phased out in the EU in 2017. Continuous HR cropping and the intensive use of glyphosate over the last 20 years has led to the appearance of at least 34 glyphosate-resistant weed species infesting millions of farmland hectares worldwide. To avoid resistance development in weeds, integrated weed management has been recommended. Conversely, companies develop transgenic crops carrying multiple HR genes, including genes that confer resistance to other herbicides, e.g. synthetic auxins or ALS-inhibitors. However, a number of hard to control weeds is already resistant to these herbicides. Experience with HR crop systems over several years shows that broad-spectrum herbicide application further decreases diversity and abundance of wild plants, in particular of broad-leaf plants, and impacts arthropod fauna and other farmland animals. Thus, adverse impacts of HR crops on biodiversity should be expected and are indeed very hard to avoid. For that reason, and in order to comply with international agreements to protect and enhance biodiversity, agriculture needs to focus on practices that are more environmentally friendly, including a reduction in pesticide use.

The present review is a condensation and update of a comprehensive technical report which was previously published by the German Federal Agency for Nature Conservation BfN, the Austrian Environment Agency EAA, and the Swiss Federal Office for the Environment FOEN (Tappeser et al. 2014). Further on and based on the technical report, a subset of members of the Interest Group GMO within the EPA- and ENCA networks^[[1]](#footnote-2)^, drafted a position paper which contains key messages regarding environmental impacts of the cultivation of genetically modified herbicide-resistant plants^[[2]](#footnote-3)^. Acting upon the key messages should improve the scope of the current environmental risk assessment of these plants. The position paper was recently addressed to relevant EU bodies with the aim to ensure adequate protection of the environment in the future.

# Review

## Introduction

There is scientific consent that biodiversity is endangered and its protection is urgent (e.g. Rockström et al. 2009). For this reason, conservation of biodiversity has received increased attention and has become an important issue of international and environmental policies. The term biodiversity, used for the variability among living organisms from all sources including terrestrial, marine and other aquatic ecosystems, and the ecological complexes of which they are part, includes diversity within species, between species, and of ecosystems (CBD, Article 2. Use of Terms)^[[3]](#footnote-4)^.

Biodiversity in agricultural landscapes can be characterized by composition (which and how many species/genotypes), structure (dominance), and function, where composition and structure can both affect its function (Duelli 1997, Büchs et al. 2003). Intensive high-input farming affects the diversity and abundance of the within-field weed flora (Hawes et al. 2010) and is one of the drivers of ongoing biodiversity losses in agricultural landscapes (Krebs et al. 1999, Robinson and Sutherland 2002, Foley et al. 2011).

## Agreements and regulations covering biodiversity protection

The protection and conservation of biodiversity has become an important issue of international and environmental policies for more than two decades. In 1992, the Rio Earth Summit agreed on the Convention on Biological Diversity (CBD) that aims at conservation of biodiversity, sustainable use of its components, and both access to genetic resources and sharing of the benefits arising out of their utilization (the Convention entered into force in 1993). In 2000, all United Nations (UN) member states and important international organizations agreed on 8 Millennium Development Goals (MDG) to be achieved by 2015, among them the goal No. 7 “to ensure environmental sustainability and to reduce biodiversity loss”. The TEEB (The Economics of Ecosystems and Biodiversity) initiative of the G8+5 Group from 2007 sought to promote a better understanding of the true economic value of ecosystem services and to contribute to more effective policies for biodiversity protection (TEEB 2008). In 2010, the UN General Assembly declared 2011-2020 the United Nations Decade on Biodiversity and released the Strategic Plan for Biodiversity 2011-2020 which aims at stopping the loss of biodiversity, while finding out the underlying causes for it, including production and consumption patterns. To achieve these goals, countries should develop national strategies and action plans. Such action plans have been implemented in a range of countries (EC 2012).

A supplementary agreement to the CBD is the Cartagena Protocol on Biosafety (CPB), adopted by the Parties to the CBD in 2000 and entering into force in 2003, with the aim to protect biological diversity from the potential risks posed by living modified organisms (LMOs)^^[[4]](#footnote-5)^^. The Protocol established a Biosafety Clearing House to facilitate information exchange on LMOs and procedures to ensure that countries can make informed decisions before they agree to the import of LMOs (advance informed agreement AIA). Actually, 195 nations plus the EU are Parties to the CBD and 169 plus the EU to the Cartagena Protocol.

In the EU, the deliberate release into the environment of genetically modified organisms (GMOs) is regulated by the Directive 2001/18/EC and its amendment, the Directive (EU) 2015/412. Referring to the precautionary principle, the Directive 2001/18/EC aims at the protection of human and animal health and the environment and at control of risks from such releases. According to this Directive, potential cumulative long-term effects of GMO releases have to be monitored and the diversity of European ecosystems has to be taken into account. In the course of the environmental risk assessment (Annex II), intended and unintended as well as cumulative long-term effects relevant to the release and the placing on the market of GMOs have to be considered comprehensively. This is in terms of human health and the environment, including inter alia flora and fauna, soil fertility, soil degradation of organic material, the feed/food chain, biological diversity, animal health, and resistance problems in relation to antibiotics.

## Herbicide-resistant crops

Herbicide-resistant (HR)^[[5]](#footnote-6)^ crops will help to further intensify farming and increase pressure on biodiversity. Although effects of genetically modified (GM) HR plants may apply also to non-GM HR plants, such as Clearfield® crops (Tan et al. 2005), and impacts on biodiversity are linked to the introduction of new crops for intensive management (Sutherland et al. 2006), the wide in-crop use of broad-spectrum herbicides such as glyphosate and glufosinate was only made possible by genetic engineering. GM crops resistant to these herbicides have first been cultivated commercially in the 1990’s (Green and Castle 2010). HR crop technology comes as a package consisting of the HR crop plus at least one complementary herbicide. The technology allows for a changed herbicide use in terms of application rate, dosage and/or crop life stage, compared to other cropping systems. According to the Council of the European Union (2008), the potential consequences for the environment of changes in the use of herbicides caused by transgenic HR plants have to be studied and the competent authorities involved in the implementation of the Directive 2001/18/EC and of the Directive on pesticides 91/414/EC (replaced by Regulation (EC) 1107/2009) should co-ordinate their action as far as possible.

Most HR crops placed on the market are resistant to either glyphosate (often called RoundupReady RR-crops) or glufosinate (also known as LibertyLink LL-crops), and increasingly both traits are combined in one crop, especially in maize and cotton^^[[6]](#footnote-7)^^. GM crops with resistance to other herbicides such as imidazolinone, sulfonylurea, dicamba, 2,4-D^^[[7]](#footnote-8)^^, HPPD^^[[8]](#footnote-9)^^- and ALS^[[9]](#footnote-10)^-inhibitors are under development (Green 2014) or already on the market, and quite often these traits are stacked with glyphosate and/or glufosinate resistance^[[10]](#footnote-11)^. Various cotton, maize, and soybean stacks are no longer considered a regulated article under USDA regulations (USDA 2015). Another strategy that is pursued for HR crops is the development of plants which are highly resistant towards glyphosate (see below).

The organic acid glyphosate inhibits 5-enolpyruvylshikimate-3-phosphate synthase (EPSPS), an enzyme of the shikimate pathway for biosynthesis of aromatic amino acids (phenylalanine, tryptophan and tyrosine) and phenolics. The enzyme is present in plants and microorganisms, but not in human or animal cells (OECD 1999a). Glufosinate ammonium is an equimolar, racemic mixture of the D- and L-isomers of phosphinothricin (PPT). L-PPT glufosinate inhibits plant glutamine synthetase, leading to the accumulation of lethal levels of ammonia (OECD 1999b). Glyphosate and glufosinate use are not unique to HR cropping systems, but they can be used in HR-crops at other application rates, dosages, and/or crop life stages, compared to other cropping systems.

Most glyphosate-resistant crops contain *epsps* genes from *Agrobacterium* spp. encoding an EPSPS protein that is insensitive to glyphosate. Others contain additionally the *gox* gene from *Ochrobactrum anthropi* encoding the glyphosate-degrading enzyme glyphosate oxidoreductase (GOX). The more recently used *gat* gene confers glyphosate resistance by expression of glyphosate acetyltransferase (GAT), an enzyme that modifies glyphosate. Another recent strategy to fight glyphosate-resistant weeds is the development of plants that are highly resistant to glyphosate due to the co-expression of *gat* and *epsps* genes (Dun et al. 2014, Guo et al. 2015).

The glufosinate-resistant crops have been transformed with one of the two bacterial genes *pat* or *bar* from *Streptomyces* spp. Both genes encode the enzyme phosphinothricin acetyl transferase (PAT) which detoxifies L-PPT thereby conferring resistance to glufosinate (L-PPT).

HR GM crops with resistance to further herbicides were developed. The *gm-hra* gene is a modified soybean *als* gene and confers resistance to ALS-inhibitors (USDA 2007). The *aad-1* and *aad-12* genes, expressed in corn, cotton, and soybean (USDA 2014), encode aryloxyalkanoate dioxygenase (AAD) proteins that degrade 2,4-D and can act on other herbicides as well, such as ACCase inhibitors^[[11]](#footnote-12)^ or other synthetic auxins (Wright et al. 2010). The dicamba mono-oxygenase (DMO) derived from the bacterium *Stenotrophomonas maltophilia* demethylates dicamba, rendering it inactive in GM soybean and GM cotton (APHIS 2014).

Many transgenic HR crop species have been globally tested in field experiments, but to date only four are widely grown commercially since the late 1990s: maize, cotton, canola, and soybean (Brookes and Barfoot 2015a). In 2013, of the 175.2 million ha global GM crop area, about 57% (99.4 million ha) were planted with HR varieties and another 27% (47 million ha) with crops with stacked traits (basically HR/insect resistance stacks) (James 2013). Hence, 84% of the GM crops carried herbicide resistance genes (146.4 million ha). This amounts to 45.7% of the global area of these four crops (320 million ha).

In 2013, HR soybean was grown on 78.7 million ha (Brookes and Barfoot 2015a), making up about four fifths of the global soybean area and 46.1% of the global GM crop area. It is the dominant GM crop grown commercially in countries such as USA (>90% of all soybean is HR), Argentina, Brazil, Paraguay, Canada, Uruguay, Bolivia, South Africa, Mexico, Chile, and Costa Rica. All GM canola is herbicide-resistant, and in 2013 it represented about 24% of the global canola area (34 million ha)^[[12]](#footnote-13)^. Herbicide resistance is also an important trait in cotton and maize, where it is often combined with insect resistance genes. In the US, HR crops such as alfalfa, sugar beet, creeping bentgrass, and rice are already deregulated and on the market or pending for deregulation (USDA 2015).

## Yields of HR crops

Yield differences of HR relative to conventional crops may be due to various reasons, such as scale and region of growing, site and size of farms, soil, climate, tillage system, weed abundance, varieties, crop management, weed control practice, farmer skills, and the education of the farm operators (Zentner et al. 2002). When comparing yields between HR and conventional varieties, their respective genetic backgrounds have to be taken into account (Holtzapffel et al. 2008). In some cases, herbicide-resistance genes may exert pleiotropic harmful effects on yield (Darmency 2012). Reviewing data about the agronomic performance of GM crops, Areal et al. (2013) concluded that although GM crops, in general, perform better than conventional counterparts in agronomic and economic (gross margin) terms, results on the yield performance of HR crops vary. While, in general, the effect of HR seeds on yields is mixed, HR traits stacked with insect resistance are reported to have higher yields (Fernandez-Cornjeo et al. 2014).

In comparison to conventional varieties, yields of Canadian HR canola varieties were lower, the same, or higher (Phillips 2003, Cathcart et al. 2006), so it seems that there is no direct correlation between the HR trait and yield. Glyphosate-resistant canola lines may show a slight yield penalty, whereas glufosinate-resistant canola cultivars were amongst the highest yielding due to intensive breeding efforts in the past two decades (Beckie 2013).

In glyphosate-resistant soybean, earlier studies have repeatedly found a yield reduction, but more recent studies show little yield difference, although subtle differences in quality-related traits may be observed (Beckie 2013). The yield drag that has been observed (Elmore et al. 2001) might be due to (i) the present resistance gene in first generation HR lines RR 40-3-2, (ii) reduced nodular nitrogen fixation upon glyphosate application and/or (iii) a weaker defence response (King et al. 2001). The applied glyphosate rather than the genetic modification affected nodule number and mass which have been correlated with nitrogen fixation (King and Purcell 2001, Powell et al. 2007).

The second generation HR soybean (RR2Y, MON 89788) is claimed to have a yield increase, compared to line RR 40-3-2, likely due to a superior recipient line plus a newer insertion method to avoid the yield drag (Gurian-Sherman 2009). However, when tested in the greenhouse, different cultivars of RR2Y performed less well than RR 40-3-2 (Zobiole et al. 2010). Data from more than 10 years of US HR soybean production show that HR crop yields are, on average, not higher and sometimes lower than yields of conventional varieties (Gurian-Sherman 2009). Different cultivars of first and second generation RoundupReady soybean have been reported to exhibit the symptom of “yellow flashing”, which is a bleaching of leaves that occurs when plants are treated with Roundup, even at labelled rates (Guo et al. 2015). “Yellow flashing” is thought to derive from the increase of shikimic acid in plants and accompanied by a decreased chlorophyll content and reduced photosynthesis. It affects nutrient uptake and results in reduced grain yield (Zobiole et al. 2012, Guo et al. 2015).

Genotype by environment interactions may explain contradictory results for glyphosate-resistant corn cultivars (Beckie 2013). Under average- to low-yield environments, glyphosate-resistant corn yielded more than conventional systems, but less under high-yield environments (Thelen and Penner 2007). Reviewing data of one or two year field studies in five states of the USA, Gurian-Sherman (2009) did not find a consistent yield advantage over conventional systems for HR corn, a finding that was confirmed by Heinemann et al. (2014) who compared GM corn in the US (where 85% of corn carry HR traits, Fernandez-Cornejo et al. 2014) with conventional varieties in Europe.

According to Khan (2015), who reviewed the adoption of glyphosate-resistant sugar beet in the USA since 2008, conventional and glyphosate-resistant sugar beet varieties produced yields that resulted in similar recoverable sucrose in Minnesota and North Dakota. This came unexpected since research studies indicated otherwise because of better weed control in GM sugar beet. Further yield comparisons in the field were not possible because nearly 100% of fields were planted to HR sugar beet after 2008. During the last years, sugar beet yields increased slowly both in the USA and in Europe, where only conventional sugar beet varieties are grown, possibly due to favourable environmental conditions. Therefore, yield increase in the USA should not be attributed to the HR trait.

## Eco-toxicological attributes of complementary herbicides

For herbicides, specific legal frameworks regulating the approval procedures and assessment criteria are established, varying to some degree in different countries (e.g. Regulation (EC) 1107/2009 and Regulation 540/2011). While glufosinate, due to its reproductive toxicity, is expected to be phased out in the EU in 2017 (EC 2011), glyphosate, authorized in 2002, is in the process of re-evaluation (EC 2010). Due to the adoption of HR crops, glyphosate is today the herbicide most widely used in the world, applied on millions of hectares of glyphosate-resistant crops and increasingly on non-HR crops (e.g. for desiccation purposes) and in non-agricultural settings. In light of the great number of glyphosate-resistant crops that are authorized or in the pipeline, glyphosate most likely will remain one of the most used herbicides.

**Glyphosate**

Glyphosate (C_3_H_8_NO_5_P; N-(phosphonomethyl)glycine), a polar, water soluble organic acid, is a potent chelator that easily binds divalent cations (e.g. Ca, Mg, Mn, Fe) and forms stable complexes (Toy and Uhing 1964, Cakmak et al. 2009). In addition to the active ingredient (a.i.) that can be present in various concentrations, herbicides usually contain adjuvants or surfactants that facilitate penetration of the active ingredient through the waxy surfaces of the treated plants. The best known glyphosate containing herbicides, the Roundup product line, often contain as a surfactant polyethoxylated tallow amine (POEA), a complex mixture of di-ethoxylates of tallow amines characterized by their oxide/tallow amine ratio (typically 15% or less of the final formulation). POEA is significantly more toxic than glyphosate (Cox and Surgan 2006) and more so in alkaline than in acid water (Diamond and Durkin 1997). The toxicity of formulations to human cells varies considerably, depending on the concentration (and homologue) of POEA (Mesnage et al. 2013). Data from toxicity studies performed with glyphosate alone over short periods of time may thus conceal adverse effects of the herbicides. In addition, toxicology studies involving one pesticide at a time may not be appropriate to detect combined effects of exposure to multiple pesticides (Relyea and Hoverman 2006) and may miss indirect effects (Preston 2002).

Glyphosate degradation is reported to be rapid (half-lives up to 130 days, sometimes 240 days) (Borggard and Gimsing 2008), its main metabolite aminomethylphosphonic acid (AMPA) degrades more slowly. Although thought to be non-mobile, due to the strong adsorption to soil particles (Giesy et al. 2000), glyphosate and AMPA reach aquatic systems, resulting in surface water concentrations in the μg/L to mg/L range (up to 1700 μg/L glyphosate and 35 µg/L AMPA, respectively, in US pond water) (WHO 2005). Both substances are frequently and widely found in US soils, surface water, and precipitation (Battaglin et al. 2014) and are transported to marine systems, as shown by Skeff et al. (2015). Groundwater can be reached too (Sanchis et al. 2011). Recently, the widespread occurrence of POEA and the persistence of POEA homologues in US agricultural soils have been reported (Tush and Meyer 2016).

As mentioned before, glyphosate inhibits the enzyme EPSPS of the shikimate pathway. Disruption of this pathway inhibits the biosynthesis of aromatic amino acids and thereby proteins, which leads ultimately to the plant’s death (OECD 1999a). It also leads to a lack of phenolics, including defen**c**e molecules (e.g. phytoalexins), lignin derivatives, and salicylic acid that functions as signal molecule (Powell and Swanton 2008). In addition, glyphosate impacts micronutrient uptake and transport in plants (Eker et al. 2006, Cakmak et al. 2009). Even low levels of residual glyphosate in soil impede the availability and uptake of Mn, Fe, Cu, and Zn by plants. An undersupply of nutrients can reduce disease resistance and plant growth, thus increasing the herbicidal activity (Johal and Huber 2009).

In soils where RoundupReady crops are grown, high concentrations of glyphosate have been recorded, up to 1500 µg/kg (1.5 ppm) glyphosate and 2250 µg/kg (2.25 ppm) AMPA in Argentina (Aparicio et al. 2013). Glyphosate impacts the composition of the soil microflora, suppressing some soil microorganisms while favouring others (Roslycky 1982, Kremer and Means 2009). This is likely linked to varying sensitivities of bacterial EPSPS enzymes to glyphosate (Clair et al. 2012). Studies based on standardised tests found no long-term effects on soil microorganisms, even if exceeding maximum application rates (Cerdeira and Duke 2006), whereas in long-term studies shifts in composition and activity of microorganism populations have been observed (Kremer and Means 2009, Zobiole et al. 2011a). For instance, beneficial fluorescent pseudomonads, associated with antagonism of fungal pathogens and manganese reduction (to Mn2+ that is taken up by plants) were reduced in the rhizosphere of RoundupReady crops. In the RoundupReady soybean system, nitrogen fixation and/or assimilation can potentially be reduced (Means et al. 2007), in particular at above label use rates and under soil moisture stress (Zablotowicz and Reddy 2007). Negative impacts of increasing glyphosate rates on nodulation, nutrient accumulation and other growth characteristics of first and second generation RR soybean**^[[13]](#footnote-14)^** have been reported (Zobiole et al. 2011b, Zobiole et al. 2012, Guo et al. 2015).

Glyphosate impacts on fungi vary, depending on study sites, species, pathogen inoculum, the timing of herbicide application, soil properties, and tillage (Powell and Swanton 2008, Sanyal and Shrestha 2008, Kortekamp 2011). Some fungi seem to be sensitive, e.g. mycorrhizal fungi (Kremer and Means 2009, Druille et al. 2013), others, including rust and blight fungi, can increase under glyphosate application. Root exudates of glyphosate-treated RR soybeans may favour growth of pathogenic Fusarium fungi (Kremer et al. 2005). Roots of glyphosate-treated RR soybeans and RR maize had from two to ten times higher Fusarium numbers, compared to untreated plants or plants treated with conventional herbicides (Kremer and Means (2009). Roundup may affect entomopathogenic fungi that combat harmful insects (Morjan et al. 2002).

Glyphosate (without surfactants) and the metabolite AMPA are said to be of low toxicity to aquatic organisms such as invertebrates and fish (WHO 1994, Giesy et al. 2000). Phytoplankton, however, may be affected by glyphosate/Roundup, as total phytoplankton decreased and cyanobacteria increased in abundance (Pérez et al. 2007, Vera et al. 2010). Cyanobacteria are remarkably tolerant to glyphosate, possibly due to an insensitive form of EPSPS and/or the ability to metabolize it (Forlani et al. 2008). Should phosphate containing glyphosate add to the phosphorous load of surface waters, harmful cyanobacteria blooms might be favoured. The natural nitrifying community may be affected if glyphosate concentrations in the water column increase, especially during bacterial growth (Sanders and Lassen 2015).

Some of the surfactants used in glyphosate formulations are significantly more toxic than the active ingredient, in particular for aquatic organisms (Brausch and Smith 2007). The toxicity of formulated products to aquatic organisms differs, due also to highly variable sensitivity of individual species (Tsui and Chu 2004). Reported LC50 values for glyphosate formulations (in the mg/L range) for a green alga, *Daphnia magna*, and carp vary considerably (up to 370 fold) (WHO 1994, Durkin 2003). In studies where *D. magna* were fed glyphosate residues for their whole life cycle, growth, reproductive maturity and offspring number were impaired (Cuhra et al. 2015). Environmentally relevant levels of exposure to both glyphosate and Roundup have led to major changes in the liver transcriptome of brown trout, reflective of oxidative stress and cellular stress (Uren Webster and Santos 2015). Simultaneous exposure to glyphosate-based herbicides and other stressors, not uncommon in nature (Relyea 2005), can induce or increase adverse impacts on fish (Kelly et al. 2010) and amphibians (Jones et al. 2011).

Amphibians are particularly at risk to be exposed to glyphosate-based products, since shallow temporary ponds, often essential to their life cycles, are areas where pollutants can accumulate without substantial dilution (Mann et al. 2003). Early data suggested that Roundup is at best moderately toxic to amphibians and glyphosate non-toxic to slightly toxic (Giesy et al. 2000). More recent data led Plötner and Matschke (2012) to conclude that glyphosate is itself toxic to amphibians, and surfactants such as POEA are even more so: sub-lethal concentrations of glyphosate and glyphosate-based herbicides can cause abnormal behaviour, teratogenic effects and developmental failures. In addition, reduced growth of algae and aquatic plants could limit the food supply for tadpoles. In case glyphosate impairs the microbial communities of their skin, amphibians may become more susceptible to parasites and pathogens. Adult and juvenile amphibians may also come into contact with glyphosate-based herbicides, e.g. when there is a temporal coincidence of glyphosate application and migration (Wagner and Lötters 2013). As virtually nothing is known about environmental concentrations of the surfactants, glyphosate levels can only be seen as approximations for contamination with glyphosate-based herbicides. Therefore, better monitoring of both amphibian populations and contamination of habitats with glyphosate-based herbicides is important (Wagner and Lötters 2013, Wagner et al. 2013).

In laboratory studies, Roundup was found to be harmless to most of the tested arthropods, among them beneficial land predators and parasites, but harmful to others (Hassan et al. 1988, EC 2002). The mortality was reduced when exposed to the glyphosate salt only. Formulated products have been reported to be toxic to predatory mites, moderately toxic to some beneficial spiders and (parasitic) wasps, and of low toxicity to earthworms (CTB 2002). When exposed to glyphosate for a longer time (100 days), the growth of an earthworm species was severely affected (Springett and Gray 1992). Locomotor activity of earthworms might be altered, too, potentially compromising their survival (Verrell and Buskirk 2004). In addition, glyphosate application reduced the number and mass of casts and reproductive success of earthworm species that inhabit agroecosystems (Gaupp-Berghausen et al. 2015).

Although glyphosate is supposed to be practically non-toxic to honeybees (Monsanto Canada 2002), Herbert et al. (2014) observed negative effects of sub-lethal concentrations on honeybee appetite behaviour and learning. Exposure to glyphosate doses commonly found in agriculture also impairs the cognitive capacities of forager bees needed to retrieve and integrate spatial information for a successful return to the hive (Balbuena et al. 2015).

Acute toxicity of glyphosate to mammals is lower relative to other herbicides. HR soybean cropping is therefore supposed to be more environmentally friendly than conventional systems, based on LD50 indicators (Nelson and Bullock 2003). Glyphosate-based herbicides, however, have been reported to be toxic to human and rat cells, impact chromosomes and organelle membranes, act as endocrine disruptors, and lead to significant changes in the transcriptome of rat liver and kidney cells (Mesnage et al. 2013, 2015, Malatesta et al. 2008, Monroy et al. 2005), sometimes in concentrations at or below the recommended values for agricultural use (Benachour and Séralini 2009). Séralini et al. (2012) reported that in a 2-year study rats fed with Roundup-treated HR maize (event NK603), untreated NK603 maize, or Roundup-containing drinking water, showed more severe effects than control animals fed with the nearest isogenic non-GM maize line. In the course of the scientific debate about the significance of these findings (Hammond et al. 2013, Séralini et al. 2013 and others**^[[14]](#footnote-15)^**) the journal editor retracted the study referring to inconclusive data (Hayes 2013a, b). The scientific debate, however, is still going on (Meyer and Hilbeck 2013, Loening 2015) and the original study has been republished (Séralini et al. 2014).

The susceptibility of bacteria (*Escherichia coli* and *Salmonella enterica* serovar Typhimurium) to antibiotics has been decreased, if simultaneously exposed to the herbicides glyphosate, dicamba or 2,4-D (Kurenbach et al. 2015). As glyphosate may impact microorganisms in the human and animal gastrointestinal tract differently, with pathogenic bacteria species (e.g. *Salmonella* and *Clostridium*) being less sensitive to glyphosate than beneficial bacteria (e.g. lactic acid bacteria like lactobacilli, lactococci and enterococci), as shown for poultry microbiota in vitro, the microbial community in the gastrointestinal tract could be negatively affected (Shehata et al. 2013). In addition, an astoundingly correlated increase of the cultivation of herbicide resistant crops and diseases related to the digestive system in humans was shown by Swanson et al. (2014). For this reason, studies on glyphosate effects on the gut microbiome of other species are needed.

Studying potential effects of very low amounts of Roundup Original® on embryos of the African clawed frog *Xenopus laevis* and chicken, Paganelli et al. (2010) reported they had found direct negative effects on embryonic development (mainly eye and head defects), presumably caused by glyphosate itself, rather than by a surfactant or other components of the commercial formulation. The teratogenic effects observed after injection have been linked to interference of glyphosate with retinoic acid signalling that plays an important role in gene regulation during early vertebrate development. After reviewing data about potential health effects, Antoniou et al. (2012) called for a new and transparent re-examination of toxicity data of glyphosate and its commercial formulations. The German competent authority, in charge of writing the report on glyphosate in the course of the EU renewal process, did not find indications for risks to human health, but suggested to improve risk management for the protection of biodiversity (BVL 2014). In contrast, the International Agency for Research on Cancer (IARC) concluded in a recent report that glyphosate is probably carcinogenic to humans (IARC 2015). When mandated by the European Commission to consider IARC’s conclusion, EFSA (2015) identified some data gaps but argued that, based on its own calculations about glyphosate doses humans may be exposed to, glyphosate is unlikely to pose a carcinogenic hazard to humans. The current concerns over the use of glyphosate-based herbicides are summarised in a recent paper (Myers et al. 2016), which concludes that glyphosate-based herbicides should be prioritised for further toxicological evaluation and for biomonitoring studies.

Glufosinate ammonium

Glufosinate ammonium is an equimolar, racemic mixture of the D- and L-isomers of phosphinothricin (PPT). L-PPT Glufosinate inhibits glutamine synthetase of susceptible plants and results in the accumulation of lethal levels of ammonia (OECD 1999b). Less data on eco-toxicity of glufosinate is available compared to glyphosate, presumably due to the significantly lower use of glufosinate. Glufosinate, as formulated product, is known to be (slightly) toxic to fish and aquatic invertebrates. Published EC50 values for formula (the same or different products) vary from 0.5 - 78 mg/l (Dorn et al. 1992). In field experiments, concentrations inducing a 20% (EC20) and 50% (EC50) reduction in abundance of various zooplankton taxa ranged from 0.03 - 0.16 mg/L and from 0.12 - 0.5 mg/L glufosinate, respectively (Faber et al. 1998). As drift events can lead to 0.25 mg/L glufosinate (formulated product) (Dorn et al. 1992), significant negative effects can be anticipated at environmentally relevant concentrations.

Glufosinate has been shown to suppress some soil microorganisms, whereas others exhibited tolerance (Ahmad and Malloch 1995). Of fungal isolates, the plant pathogen *Verticilium alboatrum* was among the most resistant, while mycoparasitic *Trichoderma* species were among the most sensitive. Some fungal pathogens seem to be reduced by glufosinate, potentially due to inhibition of glutamine synthetase, similar to the inhibition in plants (Kortekamp 2011). Glufosinate is harmful to spiders (Dorn et al. 1992) and may also impact predatory insects and mites (Ahn et al. 2001). Insecticidal activity on a skipper butterfly through glutamine depletion has been described (Kutlesa and Caveney 2001).

Glufosinate ammonium has the potential to induce severe reproductive and developmental toxicity seen as pre- and post-implantation losses, vaginal bleedings, abortions, and dead foetuses in rats and premature deliveries, abortions, and dead foetuses in rabbits (EFSA 2005). Because of its reproductive toxicity, use of glufosinate will be phased out in the EU by September 2017 (EC 2011). In other countries, however, glufosinate use may not be discontinued as glufosinate-resistant crops are increasingly grown in reaction to the ever greater number of glyphosate-resistant weeds. In the US for instance, the area of glufosinate-resistant soybean has increased three-fold, to a still low 3.9 percentage of HR crops (USDA 2014). More recently, several glufosinate-resistant crop lines have been deregulated in the US (USDA 2015).

Other herbicides

The increasing use of “old” herbicides such as synthetic auxins, expected in the course ofUS deregulation of corn and soybean resistant to 2,4-D or dicamba, raises serious concerns. Synthetic analogues of the plant hormone auxin cause uncontrolled and disorganized plant growth finally killing sensitive plants, e.g. broad-leaf weeds. The herbicide 2,4-D is 75 times and dicamba 400 times more toxic to broadleaf plants than glyphosate (Mortensen et al. 2012). As both herbicides are highly volatile, the potential for damage to non-target organisms due to spray drift would increase significantly. Sensitive crops, vegetables, ornamentals, and plants in home gardens could be damaged (Johnson et al. 2012) and both plant and arthropod communities in field edges and semi-natural habitats affected (Egan et al. 2014). With transgenic plants, the risk of non-target damage may increase since a new timing window for post-emergence application is opened in late spring to midsummer when temperatures are higher and plants are actively growing (Mortensen et al. 2012). Even sub-lethal damage of non-target plants could impact arthropods and pollinators, e.g. by altering plant nutritional content or delayed flowering onset and a reduced number of flowers (Bohnenblust et al. 2013, 2015).

A new 2,4-D formulation (choline) is reported to offer ultra-low volatility, reduced drift, decreased odour, and improved handling. It is used e.g. in the product Enlist Duo that comprises both 2,4-D and glyphosate. A dicamba formulation with lower volatility, called Engenia, to be used in crops resistant to glyphosate and dicamba, is under development, too (Lingenfelter and Curran 2013). But as long as low-cost generic formulations of both 2,4-D and dicamba are available, farmers may turn to these more volatile versions for economic reasons (Johnson et al. 2012). Whether special stewardship guidelines on nozzle type, spray boom height, ground speed, wind speed, and sensitive crop buffers, among others, will help reduce adverse herbicide effects, is highly questionable (Mortensen et al. 2012), since lower volatility of a substance may reduce exposure but not toxicity and stewardship programs address resistance issues in the target organisms and not toxicity issues. Due to potential synergistic effects between the two ingredients in Enlist Duo on non-target plants, the US Environmental Protection Agency has considered taking legal action to revoke registration of this herbicide mix**^[[15]](#footnote-16)^**.

2,4-D, one of the oldest herbicides, controls broad-leaf weeds, while monocotyledonous plants such as cereals remain mostly unaffected. It is often mixed with other active ingredients and used not only on crops but also on turf and ornamentals, and other areas. It acts as a synthetic analogue of the plant hormone auxin and causes uncontrolled and disorganized plant growth finally killing sensitive plants. 2,4-D and 2,4,5-T (2,4,5-trichlorophenoxyacetic acid) each accounted for about 50% of Agent Orange, the herbicide product sprayed by the US military in the jungle in Vietnam. Agent Orange contained highly toxic impurities, includingdioxins and furans. Such impurities in actual 2,4-D containing herbicides may still be a concern, especially in herbicides manufactured outside the EU and US (Holt et al. 2010). Recently, IARC (Loomis et al. 2015) classified 2,4-D as a “possible human carcinogen”, a classification which is not shared by EFSA (2014).

## Impacts of HR crops on agricultural practice and agronomy

HR crops are often linked to reduced tillage, but adoption of conservation tillage (that can help to prevent soil erosion, reduce soil compaction and save fuel) is influenced by other factors too, such as government programs, declining costs of pre-emergence herbicides, and improvements in seeding technologies (Zentner 2002). Between 1996 and 2008, adoption of conservation tillage in soybean increased from 51% to 63%, while no-till increased by a third to 41% (USDA 2014). In Argentina, many farmers who adopted HR soybean also reduced tillage, with 42% of conventional fields and 80% of HR fields practicing reduced tillage (Qaim and Traxler 2005).

Surveys revealed that HR transgenic crops are adopted mainly as a component of agricultural practices and weed management methods. In the US, in the first years of their adoption, improved and simplified weed control was most often stated as reason, followed by cost reduction, labor reduction, no-till planting/planting flexibility, yield increase, and in some cases decreased pesticide input (Sankula et al. 2005). Price reductions for glyphosate, reduced dockage in canola, and increased flexibility, e.g. by extending the time window for spraying, were further reasons (Mauro and McLachlan 2003, EC 2000, Firbank and Forcella 2000). Labor reduction may allow generating off-farm income (Fernandez-Cornejo et al. 2014). In general, there is a strong desire to reduce production risks (Fernandez-Cornejo and Caswell 2006). In contrast, neither biodiversity nor weed resistance management have been significant considerations to farmers (EC 2000), although soybean producers also switched to HR varieties due to problems with resistant weeds (Shaner 2000). According to Green (2014), growers urgently needed glyphosate when resistant crops became available because weeds were becoming widely resistant to most commonly used selective herbicides, making weed management too complex and time consuming for large farm operations. Reasons for adoption of HR crops in South America were similar to those mentioned above (Pengue 2004). Moreover, lack of patent protection of GM seeds seems to have made the introduction of HR soybean in Argentina easier and cheaper, as seeds could be saved for planting and resale and could also enter the black market from where they were smuggled into Brazil (Schnepf 2003).

Crop rotation helps to maintain high productivity by reducing pesticide use and fertilizer input, it also can reduce pest incidence, weed infestations, and selection pressure for weed resistance to herbicides (USDA 2014). It reduces the inoculum for diseases such as grey leaf spot (*Cercospora zeae-maydis*), which can be severe in continuous no-till maize, and allows to distribute farm work more evenly than without. As glyphosate and glufosinate are perceived to have a low residual activity, carryover restrictions are low with these two herbicides. Thus in HR crops, rotation options are increased in principle, but the experience of the last years shows otherwise (Mortensen et al. 2012). In the US, soybean is most often rotated to corn, on up to 80% of acreage (USDA 2014). This implies that on very large areas rotation comprises only HR crops, with, in 2013, 93% of US soybean and 85% of US corn being herbicide-resistant (Fernandez-Cornejo et al. 2014). In Argentina, continuous HR soybean replaced about 4.6 million ha of land initially dedicated to cotton, maize, orchards, sunflower, horticulture, as well as fallow and pasture land within the first five years, leading to a noticeable homogenisation of production and landscapes (Pengue 2004).

## Weed control patterns and herbicide use

In non-HR farming with crop rotation, usually a sequence of herbicides with different modes of action or tank mixtures are applied, some of them in pre-emergence. However, HR crops allow the post-emergence application of a single herbicide with a broad activity spectrum. Whereas post-emergence weed control in conventional farming is usually 3-5 weeks after crop emergence, it can be delayed in HR crop farming (Kalaitzandonakes and Suntornpithug 2001, Dewar et al. 2000). This increases management flexibility.

Changes in overall amount of herbicides used are difficult to assess since different herbicides are applied at different rates. Also, a change in amounts does not necessarily imply a change in side-effects or number of applications (Kleter et al. 2008). Within the first years of HR crop adoption in the US, not the application frequency but the number of different herbicides (active ingredients a.i.) has been reduced, as glyphosate was frequently applied at pre- and post-emergence in HR crops replacing other herbicides (Gianessi 2008).

Brookes and Barfoot (2015b) reported that, in the US, the overall herbicide use in HR soybean has been fairly stable for the period up to 2006, but has increased since then. From 1998 to 2013, the average active ingredient (a.i.) use (kg/ha) in HR and conventional soybean, respectively, has increased by 64% and 19%, respectively. Benbrook (2009) also found that more herbicides were applied to HR crops than to conventional crops. Between 1996 and 2008 the average amount of herbicides applied to HR soybean hectares increased almost two-fold from 0.99 to 1.84 kg a.i./ha, while in conventional soybean it dropped by 60% from 1.33 to 0.54 kg a.i./ha. Mainly due to the rising reliance on glyphosate, the HR crops soybean, maize, and cotton increased herbicide use in the US by an estimated 239 million kg in the 1996-2011 period compared to what would have been used on non-HR crops, with HR soybean accounting for 70% of the total increase (Benbrook 2012a).

Global glyphosate use increased too. While from 1995 to 2014 US agricultural use of glyphosate rose nine-fold to 113.4 million kg, global use rose almost 15-fold to 747 million kg, with more than 50% accounted for by HR crops (Benbrook 2016). In Argentina, from 1996 to 2007, the number of herbicide sprays and the amounts applied per hectare increased in reduced tillage systems planted with HR soybean four-fold to 12 L/ha^[[16]](#footnote-17)^. Overall, 20 to 26 million L glyphosate were applied on RoundupReady soybean between 1996 to 1999, rising to 100 million L in 2000, 200 million L in 2007 and up to nearly 240 million L in 2011 on an area that steadily increased within the last years (Catacora-Vargas et al*.* 2012).

Based on early EU field trials, several authors deduced that in HR oilseed rape, maize, and sugar/fodder beet the number and amount of active ingredients per ha may be reduced and that later and fewer sprays than in the conventional treatments would be necessary (Phipps and Park, 2002, Dewar et al. 2005, Champion et al. 2003). Benbrook (2012b) however, projected a significant rise in total herbicide use if HR maize, HR soybean, and HR sugar beets would be grown in the EU: after fourteen years, total herbicide use (i) remains stable (i.e. 1% reduction) without HR crop adoption, (ii) increases by 72% with an unlimited HR crop adoption similar to the US and (iii) rises by 25% with targeted adoption (i.e. accompanied by resistance management commitments). With unlimited adoption, the 31% fall in use of other herbicides would be surmounted by the explosive 824% growth of glyphosate, accounting for 65% of total herbicides.

Increased weed resistance to glyphosate leads to changes in the mix, total amount, cost, and overall profile of herbicides applied to HR crops (Brookes and Barfoot 2015b). With regard to weed control, interactions between herbicides are possible: they may be both synergistic and antagonistic (Bethke et al. 2013). To control weeds, tank mixtures of glyphosate with other herbicides have been recommended (Waggoner et al. 2011) and herbicides such as atrazine, acetoclor, dicamba, 2,4-D or mixtures of them have been added to glyphosate or glufosinate-based weed control programs (Shaner 2000). In the US, in 2013 almost two thirds of RoundupReady soybean crops received an additional herbicide treatment, compared to 14% in 2006 (Brookes and Barfoot 2015b). Use of 2,4-D, for instance, increased from 2002 to 2011 by almost 40% to 29 million kg (USDA 2014). Pre-mixed formulations and new formulation technologies shall help growers to select the optimum herbicide mixtures with diverse mode of actions (Green 2014). With the advent of stacked herbicide resistance traits in transgenic crops, “old” herbicides such as 2,4-D, dicamba, ACCase- and ALS-inhibitors are coming back. USDA (2014) expects that in the US after deregulation of 2,4-D resistant soybean and corn 2,4-D amounts applied could triple by 2020, compared to 2011 levels, whereas glyphosate use would remain stable. Benbrook (2012a) even estimates 2,4-D use on corn would increase by 2019 over 30-fold from 2010 levels.

## Changes in weed susceptibility

Both non-selective herbicides glyphosate and glufosinate are effective on a wide range of annual grass and broadleaf weed species, with glyphosate showing the broader spectrum. Glyphosate is said to control over 100 weed species, glufosinate has a somewhat smaller range. As glufosinate, contrary to glyphosate, is not translocated into the root system, it is not active on perennial structures of weeds.

The simplicity and effectiveness of weed control in HR cropping systems is a main reason for adopting this technology. It can be undermined in several ways: (i) by shifts in weed communities and populations resulting from the selection pressure of the applied herbicides, (ii) by escape and proliferation of transgenic plants as weedy volunteers, and (iii) by hybridization with – and HR-gene introgression into – related weedy species.

## Selection of resistance and weed shifts

Due to the reliance on herbicides for weed control and their increased use, the number of weeds resistant to various modes of action rose steeply within the last decades. HR cropping is no exception to this rule, as weeds will be under higher selection pressure from fewer herbicidal modes of action applied several times during the growing season, in contrast to the previous situation when growers used selective herbicides.

In early 2016 a total of 249 herbicide-resistant weed species with 464 biotypes have been recorded. These resistant weed biotypes occupy hundreds of thousands of fields worldwide, and many of them are resistant to more than one herbicide mode of action (Heap 2016), with some being resistant to more than five (Anonymous 2014). Weeds can resist herbicides through several mechanisms, including target site insensitivity, overproduction of the target protein, herbicide detoxification, reduced herbicide entry and translocation, and changes in their intracellular accumulation. Herbicide resistance in different weed populations may occur due to spread from a few initial sites, through outcrossing or because it evolved independently several times (McNaughton et al. 2005, Zelaya et al. 2007). Also, resistant seeds can be transported over large distances, through e.g. farm equipment, cars, animals, wind, and floods (Norsworthy et al. 2008, Ansong and Pickering 2013).

Weeds can exhibit cross resistance, i.e. one genetically-endowed mechanism conferring the ability to resist herbicides from different chemical classes, and multiple-resistance, i.e. expression of several resistance mechanisms within individuals or populations (Parrish 2015). The latter is presumed to develop through accumulation of resistance mechanisms as a result of gene flow between individuals with different resistance mechanisms or by selection following extensive use of two or more herbicides with different modes of action.

Glyphosate (and glufosinate) have long been considered to be low risk herbicides in terms of the evolution of resistant weed populations (Beckie 2006). This was attributed to the timing of application, the low occurrence of mutants, and the genetic background for glyphosate resistance (Neve et al. 2003). Other cited reasons were the chemical structure of glyphosate, its particular mode of action, its limited metabolism in plants, its fast degradation, its limited adsorption to and limited uptake from the soil, the perceived lack of soil persistence and residual activity, and its application pattern (Jasieniuk 1995, Johnson et al. 2009). Furthermore, before HR crops were introduced, glyphosate was mostly used in alternation or in combination with other herbicides reducing selection pressure to some extent (VanGessel 2001). It was regarded as a once in a life-time herbicide.

The first case of a glyphosate-resistant weed in conventional cropping system (rigid ryegrass, *Lolium rigidum*) was reported in 1996 after about 15 years of glyphosate use (Pratley et al. 1999). Already four years on, the first glyphosate-resistant weed (horseweed *Conyza canadensis*) was found in HR crops, in RoundupReady soybean in Delaware. To date, at least 34 cases of glyphosate-resistant weed species (more than 240 populations) have been confirmed, observed on millions of hectares, at many different locations and in various countries, and increasingly associated with HR crop cultivation (Heap 2016). In the US, the area infested likely exceeds 28 million ha by a sizable margin^[[17]](#footnote-18)^. Glyphosate-resistant palmer amaranth (*Amaranthus palmeri*), confirmed first in 2005, increasingly creates control problems and poses a major economic threat to US cotton production. The problem is worst in the Southern US states, with Mississippi having 7 glyphosate-resistant weed species (USDA 2014). In Argentina and Brazil, numbers of glyphosate-resistant weeds are also rising (Vila-Aiub et al. 2008, Heap 2016).

Over 50 glyphosate-resistant weed populations, belonging to 16 species, express resistance to other herbicide classes as well, e.g. to ALS inhibitors, ACCase inhibitors, PPO inhibitors, trifluralin or paraquat. Up to five resistances can be combined (Heap 2016). In 2010, the first weed population resistant to both glyphosate and glufosinate (Italian ryegrass) has been confirmed in Oregon

In Europe, although no glyphosate-resistant crops are authorized for cultivation, glyphosate use has increased significantly (e.g. in low-till agriculture and for desiccation) triggering a rise in the number of resistant weeds. 19 resistant biotypes, belonging to six species, have been found in the following EU countries: Spain, Greece, Italy, Portugal, France, the Czech Republic, and Poland. Spain is the most afflicted country, where horseweed (*C. canadensis*), Italian ryegrass (*Lolium multiflorum*), rigid ryegrass (*Lolium rigidum*), hairy fleabane (*C. bonariensis*), and Sumatran fleabane (*C. sumatrensis*) have infested hundreds of hectares (Heap 2016).

Resistant weeds can withstand up to 19-fold the glyphosate dose tolerated by herbicide sensitive plants (VanGessel 2001, Legleiter and Bradley 2008). Palmer amaranth was shown to have an LD50 (lethal dose to kill 50% of plants) up to 115-fold greater than that of sensitive biotypes (Norsworthy et al. 2008). The molecular and genetic mechanisms of resistance to glyphosate are very diverse and can co-occur (Perez-Jones and Mallory-Smith 2010, Zelaya et al. 2007, Bostamam et al. 2012, Sammons and Gaines 2014). The following mechanisms have been described: mutations in the critical amino acid sequence (target site) of the EPSPS enzyme (Kaundun et al. 2008, Simarmata and Penner 2008), increased EPSPS mRNA levels (Dinelli et al. 2008), and amplification (up to 160-fold) of the *epsps* gene (Gaines et al. 2010). Resistance may also be conferred by delayed translocation of glyphosate from the leaves to other plant parts (Preston and Wakelin 2008, Shaner 2009), by sequestration of glyphosate in plant cell vacuoles (Ge et al. 2010) or by degradation in the plant (de Carvalho et al. 2013). Glyphosate resistance in the French resistant *Lolium rigidum* population, for instance, is based on three different mechanisms: reduced absorption, reduced mobility in the plant, and a mutation in the *epsps* gene (Fernandez et al. 2015). Resistance mechanisms not based on target site mutations are considered particularly problematic, as they could favour evolution of resistance to other herbicidal modes of action (Yuan et al. 2007). The evolution of resistance may also be influenced by rhizosphere interactions (Schafer et al. 2012).

Herbicide resistance is mainly propagated by semi-dominant or dominant inheritance of single-gene mutation, but sometimes multiple genes are involved (Christoffers and Varanasi 2010). Hybridization between related weed species can help to spread resistance genes (Zelaya et al. 2007, Nandula et al. 2014). Fitness penalties may and may not occur in resistant weeds (Pedersen et al. 2007), but their probability, frequency and significance are not well understood. Target site overexpression (EPSPS overproduction in case of glyphosate) or detoxification likely has a significant cost of resistance, especially when extra gene expression is involved and constant. Such biotypes might disappear when the herbicide is changed.

As only a small share of cultivated HR crops is resistant to glufosinate, selection for glufosinate resistance seems to be low. Although weed species with lower sensitivity to glufosinate are known (Jansen et al. 2000, Champion et al. 2003, Heard et al. 2003b), glufosinate-resistant weed biotypes have been recorded only recently. The first two species are goosegrass (*Eleusine indica*, 2 biotypes) in Malaysia (2009) and Italian ryegrass (*Lolium multiflorum*) in Oregon (2010), the latter and one goosegrass biotype are also resistant to glyphosate (Heap 2016). The Oregon biotype requires 2.8-times higher glufosinate rates to reduce growth by 50%, caused by a single amino acid exchange in the target enzyme glutamine synthetase (Avila-Garcia et al. 2012).

The increased glyphosate use in farming has promoted species shift among the weed flora (Reddy and Norsworthy 2010), since less sensitive species and populations can survive sprayings and subsequently grow and spread, whereas more sensitive species disappear. Weed species may also avoid glyphosate by late-season or continual emergence. The soil nitrogen status could impair glyphosate’s effectiveness, too: under low nitrogen, survival rates of velvetleaf (*Abutilon theophrasti*) and common lambsquarter (*C. album*) remained relatively high (Mithila et al. 2008).

In the Southern US, a major change in the prevalence of the most troublesome weed species in cotton and soybean has occurred from 1994/1995 to 2008/2009, parallel to the rapid adoption of HR crops (Webster and Nichols 2012). Several grass and broadleaf weeds are becoming problematic weeds in glyphosate-resistant crops (Johnson et al. 2009, Reddy and Norsworthy 2010). Waterhemp is not only favoured through the herbicide management in HR cropping, but also through increased no-tillage and reduced tillage practices (Nordby et al. 2007). Weed species shift has also been observed in Argentina, where after a few years of RoundupReady soybean cultivation 37 weed species have gained in significance, while only 18 species have decreased (Vitta et al. 2004).

## Resistance management

In the beginning of HR crop cultivation, resistance management was not considered to be an issue (Bradshaw et al. 1997, Ghersa et al. 2000), but this has changed later (Buhler 2002, Powles 2008). For more than a decade now, weed scientists are recommending that farmers should implement an integrated weed management approach that reduces the selection pressure placed on weeds by glyphosate. The simplest way to do so is to avoid using glyphosate as the only weed management tool and to combine and rotate a number of weed management methods from crop rotation, mechanical weeding to cover crops, intercropping, and mulching (e.g. Wolfe 2000, Buhler 2002, Beckie 2006, Vencill et al. 2012, Norsworthy et al. 2012). Total control is not required either to prevent that weeds, non-target or beneficial wild plants compete with crops for nutrient or water (Korr et al. 1996, Werner and Garbe 1998).

Despite these recommendations, continuous glyphosate-resistant cropping is common in the Americas, and farmers often simply resort to increased herbicide doses, additional applications (often both), and other herbicides (Prince et al. 2012c). They focus rather on short-term weed control than on preventive integrated pest management practices (Wilson et al. 2008, Sanyal et al. 2008, Norsworthy et al. 2012) and do not scout their fields for problematic weeds (Johnson et al. 2009). The situation has quite changed in the last years, as more farmers surveyed in 2010, compared to 2005, recognized the importance to manage glyphosate-resistant weeds. But 30% of them still did not consider such weeds to be a problem on-farm yet (Prince et al. 2012a, Prince et al. 2012b, Prince et al. 2012d). A recent questionnaire revealed that about half (49 %) of the US farmers surveyed had problems with glyphosate-resistant weeds (Fraser 2013), while in numerous southern cotton-producing states no less than two-thirds reported herbicide-resistant weeds (Zhou et al. 2015).

In soybean, among others, paraquat and synthetic auxins are recommended in tank mixtures or in rotation with glyphosate (Beckie 2006). However, more than 30 and 32 weed species, respectively, have already populations resistant to paraquat (sometimes also resistant to glyphosate) and to synthetic auxins, respectively (Heap 2016). Merely rotating herbicides for weed control may exacerbate rather than diminish resistance problems by selecting for more generalist resistance mechanisms in weeds (Neve 2007). According to experts, new herbicides will not be developed within the next few years, due to the increased development costs and the challenge to find suitable substances that comply with the stricter standards that must be met for weed efficacy and environmental and toxicological safety (Vencill et al. 2012, Service 2013, Green 2014). Industry rather tends to modify well-known active ingredients and to stay, for instance, in the class of the ALS- or ACCase-inhibitors and to recommend use of new seed free from weed seeds, rotation to other RoundupReady crops, and the occasional use of other herbicides in RoundupReady crops (Gustafson 2008). The global market for glyphosate herbicides looks very promising at least up to 2019^[[18]](#footnote-19)^.

As mentioned earlier, new solutions to control herbicide-resistant weeds shall be provided by transgenic crops that resist higher glyphosate doses or that have stacked HR traits, such as GM maize, soybean and cotton not only resistant to glyphosate and/or glufosinate, but also to 2,4-D, dicamba, ACCase inhibitors or HPPD inhibitors (Behrens et al. 2007, Bomgardner 2012, USDA 2015, Green 2014)^[[19]](#footnote-20)^. But as resistance to these herbicides is already common among weed populations (e.g. 158 weed species have populations resistant to ALS-inhibitors, Heap 2016), stacking of HR traits in transgenic crops and increased use of herbicides other than glyphosate will not reduce the selection pressure on weeds or decrease overall herbicide amounts applied.

Against this background, integrated weed management, including crop rotation, is strongly recommended and seems to be the only sensible strategy in the long-term. In a long-term comparative field evaluation, Davis et al. (2012) showed that a four year crop rotation scheme (maize-soybean-small grain + alfalfa-alfalfa) not only helped to reduce herbicide applications and fertilizer input, but also provided similar or even better yields and economic output, compared to the two-year maize-soybean rotation. Cropping systems that apply an integrated weed management (IWM) approach, including crop rotation, cover crops, competitive crop cultivars, the judicious use of tillage, and targeted herbicide applications, are indeed competitive with regard to yields and profit to systems that rely chiefly on herbicides (Mortensen et al. 2012, Schütte et al. 2004).

However, in the US, the infrastructure (i.e. experts to conduct and teach field specific integrated pest management) is diminished over the years by the widespread adoption of preventative pest management technologies such as GMO technology (Allen 2015). Examining papers published from 1995 to 2012, Harker and O’Donnovan (2013) found that although articles on non-herbicidal weed management strategies have increased, those published on chemical control still eclipse those on any other weed management method. Effectiveness and long-run economic benefits of using best management practices depend also on the adoption by nearby farmers, but incentives to better implement IWM are still lacking (Fernandez et al. 2015). In the EU, strategies for non-herbicidal weed control are funded within the 7th Framework Programme for Research (Fontanelli et al. 2015).

## Seed escape and proliferation of HR plants

Volunteers, that is to say crop plants in the field emerging from the previous crop, create problems when the following crop is a different species or a different variety of the same species. If volunteers and crops resist the same herbicide, alternative herbicides or mixtures are needed. The advent of GM crops with stacked herbicide resistance traits will make management of volunteers in rotational crops more complex (Lingenfelter and Curran 2013). While some crops are ready volunteers and easily build up feral populations in off-field habitats, others hardly act as volunteers at all (Bjerregaard et al. 1997). In general, volunteers and feral populations of non-native crops tend to have a lower chance of surviving and cause fewer problems.

Oilseed rape readily produces volunteers and feral plants, due to its high seed production, high seed losses during harvest and along transport routes, and its secondary dormancy (Thöle and Dietz-Pfeilstetter 2012). In Canada, about 6% of the crop seed yield is lost on average, which is about 20 times the normal seeding rate of 4-5 kg/ha (Gulden et al. 2003). Feral populations may result from seed immigration from neighboring fields, from seed transport or from the seed bank (Pivard et al. 2008). Knispel and Mclachlan (2009) found that escaped populations persist at large spatial and temporal scales and that anthropogenic dispersal processes play an important role. HR feral oilseed rape plants have been found along transport routes in the US (Schafer et al. 2011) and also in Switzerland and Japan, although GM plants had never been grown there (Schoenenberger and D’Andrea 2012, Schulze et al. 2014, Kawata et al. 2009). HR oilseed rape plants have been found 10 years after an experimental release, although the field had been checked regularly for volunteers to prevent seed return (D’Hertefeldt et al. 2008). The recently reported incidence of oilseed rape seed contamination by the non-approved OXY-235 variety (resistant to oxynil herbicides) in the EU might be traced back to field trials in France in the 1990’s (Devaux et al. 2008), indicating that volunteers may emerge even after almost 20 years. Volunteer and feral management should therefore be a multi-scale approach and has to extend over considerable time spans.

## HR-gene flow to volunteers, neighbouring crops or interfertile weeds

The frequency of outcrossing depends on the crop species in question and its pollination system, the distance to simultaneously flowering volunteers or relatives. Further variables are genotype, abundance and foraging behavior of pollinators, weather conditions, time of the day, and the size of pollen donor and receiving populations. Several reviews have been published, focusing on the main GM crops (Andersson and de Vicente 2010, Mallory-Smith and Zapiola 2008) or on single crop species such as oilseed rape (Hüsken and Dietz-Pfleilstetter 2007, Jørgensen et al. 2009), maize (Czarnak-Klos and Rodríguez-Cerezo 2010), rice (Lu and Snow (2005), sugar beet (Darmency et al. 2009), and soybean (Lu 2005).

Pollen flow can extend to distances over several kilometers (Rieger et al. 2002) and was found up to 26 km for oilseed rape, perhaps due to far-flying pollen beetles (Ramsay et al. 2003). As large pollen sources, such as crop fields, interact on a regional scale and tend to increase gene flow, isolation distances have to be adjusted for this factor (Shaw et al. 2006).

Novel combinations of transgenic events can be formed in the wild, as shown in Canada and the US, where HR oilseed rape volunteers have been found that most probably resulted from pollen flow between adjacently-planted resistant varieties, since they carried resistances not commercially planted (Hall et al. 2000, Knispel et al. 2008, Schafer et al. 2011).

Gene flow can also extend to weeds if they can cross with the related crop. In centres of crop origin and regions where interfertile weeds (sexually compatible weeds) are present, gene flow from crop to weeds should be taken into account. This is of particular relevance for oilseed rape (*Brassica napus*) and its close relative field mustard (*Brassica rapa*) in many regions of Europe (Jørgensen et al. 2009). As spontaneous hybridizations occurring in nature are difficult to detect and reliable data is lacking, the number of hybrids within an area can only be estimated.

Once (trans-)genes conferring herbicide resistance move into weeds, their frequency within local weed populations will increase, if selection pressure is exerted by the corresponding herbicide. Hybrids do not need to be particularly fit as long as they are able to backcross with the weedy relative, a capacity which is characteristic for many interspecific hybrids. The fitness of hybrids should be assessed species by species. But even genotypes with a lower fitness may survive if the pollen flow is steady and the pollen source is large (Gliddon 1999). Contrary to a common view, application of the complementary herbicide is not a condition for an escaped herbicide transgene to persist in nature, e.g. in wild soybeans (Guan et al. 2015). Wang et al. (2014) found that overexpression of a native EPSPS protein in rice to make crops herbicide-resistant was advantageous for weedy rice, even in the absence of the herbicide. Therefore, the new trait should also be carefully considered when assessing the fitness of hybrids.

## Agriculture and biodiversity

Agriculture both impacts biodiversity and depends on biodiversity. In particular high-input farming is a major force driving biodiversity loss and other environmental impacts beyond the “planetary boundaries” (Firbank et al. 2008, Rockström et al. 2009, Foley et al. 2011). Drivers are, among others, the low number of cropped species, reduced rotation, limited seed exchange between farms, drainage, and landscape-consolidation, and not the least, increased use of pesticides. In regions such as Europe, where a good portion of the land is farmed, it is especially important to farm in a way that allows biodiversity to thrive within farmland alongside or within crops. Agriculture also relies on ecosystem functions and services and on biodiversity. This includes pollination, biological pest control, maintenance of soil structure and fertility, nutrient cycling and hydrological services (Tscharntke et al. 2005, Power 2010, Garibaldi et al. 2011, Foley et al. 2011). Reduced biological complexity is associated with increased pest populations (Lundgren and Fausti 2015).

Weeds are commonly regarded as pests because they compete with the crop for water, light, and nutrient resources and can cause harvest or quality problems. But weeds offer considerable benefits for the agroecosystem as well: they support a range of organisms, in particular arthropods, among them decomposers, predators, pollinators, and parasitoids, providing food and shelter for them (Marshall et al. 2003). Decreasing the antagonists of pests could increase pesticide inputs to substitute them, as demonstrated by exclusion experiments (Edwards et al. 1979, Thies et al. 2011). The decline in pollinator abundance and diversity also reduces yield and quality in crops that depend on animal pollination (Nicholls and Altieri 2013, Vanbergen and The Insect Pollinators Initiative 2013). Weed diversity and abundance is strongly influenced by management practices (Hawes et al. 2010). Reduced tillage not only lowers soil erosion, but also impacts the abundance and composition of weed populations (Swanton et al. 1993). This also refers to soil-dwelling arthropod species which partly prefer less disturbance but strongly depend on dead or living plant material for food and coverage (Wardle et al. 1999, Kromp 1999, Stinner and House 1990). Herbicides reduce the density and diversity of the weed flora more effectively than mechanical weeding, though the latter is more labour intensive (Schütte 2002). Non-target impacts on plants in hedgerows and woodlots close to agricultural fields have also been observed, leading to delayed flowering and reduced seed set (Boutin et al. 2014).

Within the last decades, the diversity of associated agricultural flora and the reservoir of viable seeds in arable soils has been reduced significantly, with losses of >90% for some species (Robinson and Sutherland 2002, Marshall et al. 2003). As insects often depend on certain plants during early larval stages, each plant species may be essential for, on average, 10 - 12 insect species in northern Europe (Heydemann 1983). A decrease in associated flora and arthropod abundance and diversity affects the whole food chain including small mammals and farmland birds, the latter being major targets and important indicators of agricultural change (Ormerod and Watkinson 2000). In many countries, a massive decline of abundance and diversity of birds, in particular farmland birds, has been observed (Krebs et al. 1999, Leech 2002, Marshall et al. 2003, Guerrero et al. 2012). A time lag of about 6 years between agricultural change and the decline of farmland bird population indicates that effects of agricultural intensification on habitat quality may not become apparent for several years (Chamberlain et al. 2000).

Organic farming has a large positive effect on biodiversity with plants benefiting the most among taxonomic groups (Tuck et al. 2014). It increases abundance and diversity of the weed flora (Schütte 2003) and may support rare species (Marshall et al. 2003). Results vary (Hawes et al. 2010) and are less pronounced on locations with an already depleted soil seed bank due to long-lasting former herbicide usage. Organic wheat production, for instance, favoured broad-leaf, insect-pollinated, and legume weeds and led to similar diversity of weed species between crop fields and edges, whereas herbicide treatment particularly affected the inner-field (Romero et al. 2008). In hedgerows adjacent to organic fields, the number of flowering plant species was higher and they flowered earlier and for longer periods of time, compared to the same plants adjacent to conventional fields, providing better conditions for pollinators (Boutin et al. 2014).

## Indirect effects of HR agriculture on biodiversity

HR crop cultivation can change farming practices, e.g. crop rotation, crop planting and spacing, soil tillage, pesticide application, and use of fertilisers and thus affects the environment. Potential environmental impacts of HR cropping, be they direct or indirect, have been assessed by life-cycle assessment (Bennet et al. 2006) or bow-tie risk management (Pidgeon et al. 2007). But, as these techniques necessarily involve a certain amount of subjectivity, assumptions involved and decisions taken should be made transparent.

The broad-spectrum herbicides glyphosate and glufosinate are effective on more weed species than other currently used herbicides, and mechanical weeding, and that is necessary for crop protection and productivity. Targets of so called “improved” weed control are usually a few highly damaging weeds, but many harmless and benign wild plants are killed by the non-selective herbicides, too. Therefore, weed suppression is intensified in most crops and regions where HR crops are planted. For this reason, HR crops will likely drive agriculture farther towards monoculture and excessive weed control in agricultural environments (Dale et al. 2002). Even if highly effective, broad-spectrum herbicides were applied in lower amounts or fewer applications, as often described for the first years of HR crop adoption, there is not necessarily less damage to biodiversity.

Indications of increased loss of biodiversity have been found in the three-year Farm Scale Evaluations (FSE), where the effects of the HR cropping system on abundance and species diversity were investigated in over 60 fields split in half, selected to represent the variation of geography and intensity of management across Britain (Firbank et al. 2003a, Squire et al. 2003). The results of the FSE trials have been published in numerous peer-reviewed articles. Differences were found in weed flora between different weed management regimes (Heard et al. 2003a, Heard et al. 2003b, Firbank et al. 2003b). In HR sugar beet, HR fodder beet (both glyphosate-resistant) and HR summer oilseed rape (glufosinate-resistant), the density, biomass and seed rain were between one-third and one-sixth lower, compared to conventional management. The seed bank abundance (for 19 out of 24 species) was overall 20% lower in the three HR crops (Heard et al. 2003a, 2003b). In HR beets and oilseed rape, less species emerged than in conventional crops. Compounded over time, population densities of the field flora would be largely decreased (Heard et al. 2003b). Similar results have been found by Bohan et al. (2005), studying glufosinate application in HR winter oilseed rape. FSE findings with glufosinate-resistant maize showed more diverse weed species, compared to conventional maize sprayed with atrazine, which is highly effective on a broad range of plants. However, since 2004 atrazine is no longer approved in the EU because of groundwater contamination^^[[20]](#footnote-21)^^.

Sweet et al. (2004) deduced from the BRIGHT study that the changed herbicide management of transgenic crops did not significantly decrease plant species diversity. However, the BRIGHT study was designed to explore practical issues for farmers of growing GM crops, but not the effects on wildlife. Dewar et al. (2003) and Strandberg and Pedersen (2002) reported that weed diversity in HR crops was higher in the early season, compared to conventional management. However, as weeds hardly produced seed due to late herbicide application, the long-term effects on diversity would be negative. In a one year Canadian canola field study of different rotations with high frequencies of HR crops, the overall species diversity of weeds declined by 26% and their density was reduced by 66% (Harker et al. 2004, cited in Schütte 2005).

Drift of herbicides, in particular of non-selective herbicides, to field margins is a concern to nature conservation and biodiversity of many agricultural landscapes (Boutin et al. 2014, Orson 2002, de Snoo and van der Poll 1999, Schmitz et al. 2013, Schmitz et al. 2014a, Schmitz et al. 2014b). As field margins often harbour rare plant species, the impact of non-selective herbicides on them and on the associated fauna is of particular significance. Spray drift can also damage hedgerows and trees growing close to arable fields, these habitats being very important for arthropods and birds for food, shelter, and nesting (Roy et al. 2003). The FSE trials considered some of these habitats as well: cover of HR oilseed rape and beet crop margins was reduced significantly, compared to conventional crops, impacting seeding and flowering of wild plants. Seeding was 39% lower and flowering was reduced by 44% and 34%, respectively, whereas in HR maize cover and flowering in margins was higher, compared to atrazine-treated non-GM maize (Roy et al. 2003).

The indirect effects of plant suppression and habitat destruction are the key to invertebrate and vertebrate biodiversity. In the FSE trials, the abundance of arthropods changed in the same direction as their resources (Hawes et al. 2003). In HR-beet and oilseed rape, numbers of within-field epigeal and aerial arthropods were smaller, due to forage reductions (Haughton et al. 2003, Brooks et al. 2003), and herbivores, pollinators, and beneficial natural enemies of pests were reduced (Hawes et al. 2003). In HR soybean, less canopy arthropods and significantly less spiders and green lacewings than in conventional soybean have been observed (Buckelew et al. 2000, Jasinski et al. 2004). Other studies found no significant differences between both types of crops for pest and beneficial insects (Jackson and Pitre 2004, Morjan and Pedigo 2000). Short-term differences on collembola were attributed to resultant differences in weed cover and soil disturbance (indirect effects) but not to the use of herbicides themselves (Bitzer et al. 2002). In Canadian canola fields, wild bee abundance was highest in organic fields, followed by conventional fields and lowest in HR crops (Morandin and Winston 2005), pollination decreased with bee abundance.

Models simulating effects of planting of HR crops on a larger scale came to different results: Butler et al. (2007) predicted only limited effects on farmland birds after nationwide replacement of conventional crops by HR crops in the UK. They reasoned that species relying solely on cropped areas likely decline at their current rate, regardless of whether HR or conventional crops are grown (only improving the value of cropped areas would help). Other models predicted that HR cropping will cause a major loss of food sources for animal populations on farmland and on seed consuming farmland birds (Watkinson et al. 2000, Heard et al. 2005, Bohan et al. 2005, Gibbons et al. 2006). Amphibians may also be affected, if broad-spectrum herbicide use diminishes weed abundance and spectra, because migrating adults may have difficulties finding enough invertebrates for food (Plötner and Matschke 2012, Wagner and Lötters 2013).

Recent data from the US and Mexico indicate that, within the last decade, and in parallel to the widespread and increased adoption of HR crops, the size of the Mexican overwintering population of the migratory monarch butterfly (*Danaus plexippus*) has declined significantly (Brower et al. 2012). The rapid adoption of HR crops has led to a drastic reduction of milkweed (*Asclepias syriaca*) populations, the main food plant of monarch larvae (Pleasants et al. 2016). Milkweed plants in the Midwest, the main breeding ground of monarchs, may have declined by up to 60% (some say even 90%, Hartzler 2010) and monarch propagation by about 80% (Pleasants and Oberhauser 2013). In December 2013, an all-time low of monarchs was recorded in Mexico (Wade 2014). Lincoln Brower is cited as saying “*The monarch may also be the first sign that food webs in the U.S. Midwest are being irrevocably disrupted as a side effect of widespread planting of herbicide-tolerant crops.* *Monarchs* *are “the canary in the cornfield*.” In case HR maize and HR oilseed rape crops would be widely grown in Europe, a similar scenario has been predicted for the European butterfly Queen of Spain fritillary (*Issoria lathonia*) (Hilbeck et al. 2008).

## Aspects of sustainable agriculture

Measures to mitigate environmental effects of herbicides in conventional systems have been developed in some countries. The promotion of unsprayed field margins and in-field areas and the reduction of number and doses of applications have allowed weeds and associated biota to develop, when the seed bank is not already depleted. Similar measures have been proposed for HR crops (Pidgeon et al. 2007). But in HR beet, delayed spraying within a single season increased weed biomass only transiently and only in soils which already had a rich seed bank (Dewar et al*.* 2000, Strandberg and Pedersen 2002). In the long-term, the seed bank will be reduced (Freckleton et al. 2004). Although low-dose post-emergence application has been suggested to reduce negative impacts on weed and insect biomass (Dewar et al. 2002), patchy weed control with selective herbicides may be better for biodiversity than spraying of non-selective ones (Dzinaj et al. 1998, Lettner et al. 2001).

Farmers often rely heavily on herbicides, and do not accept other management measures readily. But to avoid evolution of resistant weeds and reduce the impact of herbicides on biodiversity, the focus should change from weed control by herbicides to integrated weed management (IWM) that uses a range of measures and does not consider a clean field to be of utmost importance. Weed research should not only address relatively quick prescriptive solutions for weed problems, such as herbicide application, but develop real IWM that integrates weed biology and ecology and implements diverse combinations of IWM systems (Harker and O’Donnovan 2013, Mulik 2015).

The overreliance of HR cropping systems on chemical weed control, often benefitting from subsidies, creates a type of farming that is suited towards low biodiversity (mono cropping) and that is most economical when herbicides can be sprayed in great quantities using specialised machinery. It discourages the use and retention of existing alternative weed management skills and is not compatible with mixed cropping systems (Quist et al. 2013). Diversification practices, however, such as cover crops, mixed cropping, intercropping, and agroforestry, help retain soil and soil moisture better than intensive cropping and improve resiliency to climate disasters (Altieri et al. 2012). In addition, integrated farming systems in which a variety of products, such as grains, fruits, vegetables, fodder and livestock, are simultaneously produced, are more productive than large conventional farms, if total output, including energy input/output, is considered rather than single crop yield per hectare (Chapell and LaValle 2011). Such yield advantages can be considerable, since polycultures, often relying on high genetic diversity, reduce losses due to weeds, insects, and diseases and make a more efficient use of water, light, and nutrients and also increase soil organic matter (Altieri et al. 2012).

Development of ecosystem services in more diverse rotations displaces the need for external synthetic inputs such as N fertilizer and herbicides to maintain crop productivity, as shown in a nine-year field study in the Central US maize production region (Davis et al. 2012). Changing the cropping system from a 2-year rotation of corn and soybean to 3-year and 4-year rotations (including forage legumes), enhanced yields of corn and soybean grain by up to 9% and reduced fertilizer application, energy use, and herbicide input significantly (88% less herbicides leading to a two hundred-fold lower freshwater toxicity). Weed control and profitability remained the same, whereas labour demand was higher. Reintegration of crop and livestock production was seen as an important principle in sustainable agriculture where system boundaries should be drawn to minimize external costs.

As pointed out by the International Assessment of Agricultural Knowledge, Science and Technology for Development (IAASTD 2009), agriculture is multifunctional^[[21]](#footnote-22)^ and serves diverse needs. But for many years, agricultural science and development have focused on delivering technologies to increase farm-level productivity rather than integrating externalities such as impacts on biodiversity and the relationship between agriculture and climate change. In view of the current challenges IAASTD concludes: *Business as usual is not an option,* and, in the brief report “Towards multifunctional agriculture for social, environmental and economic sustainability”: “*Thus, increased attention needs to be directed towards new and successful existing approaches to maintain and restore soil fertility and to maintain sustainable production through practices such as low-input resource-conserving technologies based on integrated management systems and an understanding of agro-ecology and soil science (e.g. agroforestry, conservation agriculture, organic agriculture and permaculture.”*

From the data collected and assessed, HR cropping systems seem to be no option for a sustainable agriculture that focuses also on protection of biodiversity. On the contrary, HR crops rather seem to be part of the problem.

# Conclusions

The need to protect biodiversity and stop its loss is an internationally agreed goal. Scientific data give evidence that intensive high-input farming is one of the main drivers of ongoing biodiversity loss in agricultural landscapes. Diversity and abundance of the weed flora provide relevant indicators for farmland biodiversity.

HR crops, introduced in the 1990’s, facilitate weed control for farmers and make chemical weed management more flexible. Yield increase is not the main reason for adoption of HR crops, as there has been little, if any, contribution of HR crops to increase yield. HR crops are adopted primarily due to the expected lower costs, less labour and fuel consumption.

HR cropping is associated with the use of broad-spectrum herbicides. While glufosinate, due to its reproductive toxicity, is expected to be phased out in the EU in 2017, glyphosate is presently evaluated for renewed approval in the EU. It is today the most widely used herbicide in the world. In general, eco-toxicity of glyphosate has been considered to be low, compared to some other herbicides, but data collected within the last years indicate that glyphosate-based herbicides can be toxic not only to plants, but also to other life forms, in particular to aquatic species and to amphibians. Adverse effects on the soil microflora and fauna and on plant disease resistance have been reported.

Lower herbicide use may have been a benefit in the first years of HR cropping in the US, but the trend turned around 2000 and since then, herbicide use, in particular of glyphosate, increased almost steadily. The trends are similar in other countries with HR crop cultivation, such as Argentina. Should HR crops be authorized for cultivation in the EU, a significant increase in herbicide use can be expected.

In regions where HR crops are widely adopted, mechanical weed control decreased and less crop rotation and crop diversification takes place, whereas reduced till or no-till practices expanded. There is a clear trend towards monoculture of HR crops, which enhances disease and pest pressure. Increased dependence on herbicides for weed control leads to a shift in weed species composition. Although glyphosate was not considered to be a high-risk herbicide with regard to resistance development, its intensive use has led to the appearance of at least 34 glyphosate-resistant weed species (17 dicots and 17 monocots) comprising more than 240 populations and infesting millions of hectares. These biotypes exhibit a great diversity of molecular and genetic resistance mechanisms and some of them are cross-resistant to other herbicides. Recently, two weed species resistant to glufosinate have been described as well.

To combat resistance development in weeds, weed scientists recommend that farmers should use a variety of weed management methods and not rely solely on herbicides. But fus and widespread glyphosate-resistant cropping has became common in the Americas and farmers often simply resort to higher herbicide doses and other herbicide modes of action. Increasingly, companies develop and commercialize transgenic crops with stacked HR traits, among them resistance to herbicides such as synthetic auxins or ALS-inhibitors. However, a number of hard to control weeds is already resistant to these herbicides.

In addition to herbicide-resistant weeds, control problems can also arise due to volunteers of HR crops. Oilseed rape is a particularly likely volunteer, as its small long-lived seeds are easily spilled in and outside fields and along transport routes. Volunteers and feral plants, resistant to glyphosate and glufosinate have been detected in fields and areas where HR crops have not been planted previously. Oilseed rape plants with multiple HR genes not commercially sold provide evidence of novel transgene combinations in the wild. Thus, the HR trait can spread both spatially and temporally. If outcrossing of HR crops into the same or related species occurs, more HR plants might show up. Such transfer of HR genes to wild relatives should particularly be taken into account and avoided in centres of crop origin and regions where sexually compatible and weedy hybrids occur.

The Farm Scale Evaluations have provided ample evidence that in HR systems, compared to conventional farming, weeds are removed more efficiently, leading to a further reduction of flora and fauna diversity and abundance in farmland. A prominent example in this respect may be the significant reduction in monarch butterfly populations in the US which has been linked to the widespread cultivation of HR crops in the Midwest leading to a massive loss of milkweed plants, on which monarch larvae feed.

As agricultural intensification and pesticide use are among the main drivers of biodiversity loss, agreement is required on farming practices that are more environmentally friendly and less dependent on pesticides. But the lessons learnt in HR crop adopting countries indicate that herbicide use is increasing with this technology. Therefore, it is highly questionable whether present HR systems comply with measures to stop the loss of biodiversity on farmland or can be managed in a sustainable way. From a nature protection perspective, HR crops seem to be no option for a sustainable agriculture focusing also on protecting biodiversity. To avoid further adverse impacts on biodiversity, a different approach to agriculture is clearly necessary.

# References

Ahmad, K. & Malloch, D. (1995). Interaction of soil microflora with the bioherbicide phosphinothricin. Agriculture, Ecosystems and Environment 54: 165-174.

Ahn, Y.J., Kim, Y.J. & Yoo, J.K. (2001). Toxicity of the herbicide glufosinate-ammonium to predatory insects and mites of Tetranychus urticae (Acari: Tetranychidae) under laboratory conditions. J. Econ. Entomology 94: 157-161.

Allen C. (2015). History of Pest Management in Texas and the Southern United States and How Recent Grower Adoption of Preventative Pest Management Technologies Have Diminished the Capability for IPM Delivery. Outlooks on Pest Management, 26(2): 52-55.

Altieri, M.A., Funes-Monzote, F.R. &Petersen, P. (2012). Agroecology efficient agricultural systems for smallholder farmers: contributions to food sovereignty. Agron. Sustain. Dev. 32: 1-13.

Andersson, M.S. & de Vicente, M.C. (2010). Gene flow between crops and their wild relatives. Canola, Oilseed Rape 73-123; Soybean 465-481. Baltimore, Maryland.

Anonymous (2014): A growing problem, Nature 510: 187.

Ansong, M. & Pickering, C. (2013). Are weeds hitchhiking a ride on your car? A systematic review of seed dispersal on cars. PLOS ONE 8. www.plosone.org

Antoniou, M., Habib, M.E.M., Howard, C.V., Jennings, R.C., Leifert, C., Nodari, R.O., Robinson, C. & Fagan, J. (2012). Teratogenic effects of glyphosate-based herbicides: Divergence of regulatory decisions from scientific evidence. J Environ Anal Toxicol S4:006. doi:10.4172/2161-0525.S4-006

Aparicio, V.C., De Gerónimo, E., Marino, D. Primost, J., Carriquiriborde, P., Costa, J.L. (2013). Environmental fate of glyphosate and aminomethylphosphonic acid in surface waters and soil of agricultural basins. Chemosphere 93: 1866-1873.

APHIS (2014) Final Environmental Impact Statement Monsanto’s Dicamba/Glufosinate Tolerant Cotton and Dicamba Tolerant Soybean. <https://www.aphis.usda.gov/publications/biotechnology/2014/faq_brs_final_eis_cotton_soybeans.pdf>

Areal, F.J., Riesgo, L. & Rodríguez-Cerezo, E. (2013). Economic and agronomic impact of commercialized GM crops: a meta-analysis. J. Agricultural Science 151: 7-33.

Avila-Garcia, W.V., Sanchez-Olguin, E., Hulting, A.G. & Mallory-Smith, C. (2012). Target-site mutation associated with glufosinate resistance in Italian ryegrass (Lolium perenne L. ssp. Multiflorum). Pest Manag Sci 68: 1248-1254.

Balbuena, M.S., Tison, L., Hahn, M.L., Greggers, U., Menzel, R. & Farina, W.M. (2015). Effects of sub-lethal doses of glyphosate on honeybee navigation. J Exp Biol. doi:10.1242/dev.117291.

Battaglin, W.A., Meyer, M.T., Kuivila, K.M. & Dietze, J.E. (2014). Glyphosate and its degradation product AMPA occur frequently and widely in U.S. soils, surface water, groundwater, and precipitation. J. American Water Resources Association 50: 275-289.

Beckie, H.J. (2006). Herbicide-resistant weeds: management tactics and practices. Weed Technol 20: 793-814.

Beckie, H.J. (2013). Herbicide-resistant (HR) crops in Canada: HR gene effects on yield performance. Prairie Soils & Crops Journal 6: 33-39. [www.prairiesoilsandcrops.ca](http://www.prairiesoilsandcrops.ca)

Behrens, M.R., Mutlu, N., Chakraborty, S., Dumitru, R., Zhi Jiang, W., LaVallee, B.J., Herman, P.L., Clemente, T.E. & Weeks, T.P. (2007). Dicamba resistance: enlarging and preserving biotechnology-based weed mangagement strategies. Science 316: 1185-1188.

Benachour, N. & Séralini, G.-E. (2009). Glyphosate formulations induce apoptosis and necrosis in human umiblical, embryonic, and placental cells. Chemical Research in Toxicology 22: 97–105.

Benbrook, C.M. (2009). Impacts of genetically engineered crops on pesticide use: The First Thirteen Years. http://www.organic-center.org/reportfiles/GE13YearsReport.pdf

Benbrook, C.M. (2012a). Impacts of genetically engineered crops on pesticide use in the U.S. – the first sixteen years. Env. Sciences Europe 24:24 doi:10.1186/2190-4715-24-24.

Benbrook, C.M. (2012b). Glyphosate tolerant crops in the EU. A forecast of impacts on herbicide use. <http://www.greenpeace.org/international/Global/international/publications/agriculture/2012/GI_Herb_Use_FINAL_10-18-12.pdf>

Benbrook, C.M. (2016). Trends in glyphosate herbicide use in the United States and globally. Environ Sci Eur 28: 3. Doi 10.1186/s12302-016-0070-0.

Bennett, R.M., Phipps, R.H. & Strange, A.M. (2006). An application of life-cycle assessment for environmental planning and management: the potential environmental and human health impacts of growing genetically modified herbicide-tolerant sugar beet. J Environ Planning Manag. 49: 59-74.

Bethke, R.K., Molin, W.T., Sprague, C. & Penner, D. (2013). Evaluation of the interaction between glyphosate and glufosinate. Weed Science 61: 41-47.

Bitzer, R.J., Buckelew, L.D. & Pedigo, L.P. (2002). Effects of transgenic herbicide-resistant soybean varieties and systems on surface-active springtails (Enthognatha: Collembola). Environ. Entomol. 31: 449-461.

Bjerregaard, B., Madsen, K.H. & Streibig, J.C. (1997). Herbicide resistant crops and impact of their use. Danish Environmental Protection Agency, Environmental Project No. 363. <http://www2.mst.dk/Udgiv/publications/1997/87-7810-813-6/pdf/87-7810-813-6.pdf>

Bohan, D. A., Boffey, C. W. H., Brooks, D. R., Clark, S. J., Dewar, A. M., Firbank, L. G., Haughton, A. J., Hawes, C., Heard, M. S., May, M. J. et al. (2005). Effects on weed and invertebrate abundance and diversity of herbicide management in genetically modified herbicide-tolerant winter-sown oilseed rape. Proc. R. Soc. B 272: 463-474.

Bohnenblust, E., Egan, J.F., Mortensen, D. & Tooker, J. (2013). Direct and indirect effects of the synthetic-auxin herbicide dicamba on two lepidopteran species. Environ. Entomol. 42: 586-594.

Bohnenblust, E.W., Vaudo, A.D., Egan, J.F., Mortensen, D.A. & Tooker, J.F. (2015). Effects of the herbicide dicamba on non-target plants and pollinator visitation. Environ. Toxicol. Chem. Doi: 10.1002/etc.3169.

Bomgardner, M.M. (2012). War on weeds. Chemical & Engineering News 90: 20-22.

Borggard, O.K. & Gimsing, A.L. (2008). Fate of glyphosate in soil and the possibility of leaching to ground and surface waters: a review. Pest Manag Sci 64: 441-456.

Bostamam, Y., Malone, J.M., Dolman, F.C., Boutsalis, P. & Preston, C. (2012). Rigid ryegrass (*Lolium rigidum*) populations containing a target site mutation in EPSPS and reduced glyphosate translocation are more resistant to glyphosate. Weed Science 60: 474-479.

Boutin, C., Strandberg, B., Carpenter, D., Mathiassen, S.K. & Thomas, P.J. (2014). Herbicide impact on non-target plant reproduction: What are the toxicological and ecological implications? Env. Pollution 185: 295-306.

Bradshaw, L.D., Padgette, S.R., Kimball, S.L. & Wells, B.H. (1997). Perspectives on glyphosate resistance. Weed Technol 11: 189-198.

Brausch, J.M. & Smith, P.N. (2007). Toxicity of Three Polyethoxylated Tallowamine Surfactant Formulations to Laboratory and Field Collected Fairy Shrimp, *Thamnocephalus platyurus*. Arch. Environ. Contam. Toxicol. 52: 217–221.

Brooks, D.R., Bohan, D.A., Champion, G.T., Haughton, A.J., Hawes, C., Heard, M.S., Clark, S.J., Dewar, A.M., Firbank, L.G., Perry, J.N. et al. (2003). Invertebrate responses to the management of genetically modified herbicide -tolerant and conventional spring crops. I. Soil-surface-active invertebrates. Philos Trans R Soc Lond B Biol Sci 358: 1847-1862.

Brookes, G. & Peter Barfoot, P. (2015a) Global income and production impacts of using GM crop technology 1996–2013, GM Crops & Food: Biotechnology in Agriculture and the Food Chain, 6:1, 13-46, DOI: 10.1080/21645698.2015.1022310.

Brookes, G. & Barfoot, P. (2015b). GM crops: global socio-economic and environmental impacts 1996-2013. <http://www.pgeconomics.co.uk/page/38/>

Brower, L.P., Taylor, O.R., Williams, E.H., Slayback, D.A., Zubieta, R.R. & Ramirez, M.I. (2012). Decline of monarch butterflies overwintering in Mexico: is the migratory phenomenon at risk? Insect Conservation and Diversity 5: 95-100.

Büchs, W. Harenberg, A., Zimmermann, J. & Weiß, B. (2003). Biodiversity, the ultimate agri-environmental Indicator? Potential and Limits for the Application of faunistic Elements as gradual Indicators inAgroecosystems. Agric Ecosyst Environ 98: 99-123.

Buckelew, L. D., Pedigo, L. P., Mero, H. M., Owen, M. D. K. & Tykla, G. L. (2000). Effects of Weed Management Systems on Canopy Insects in Herbicide-Resistant Soybean. J. Econ. Entomol. 93 (5): 1437-1443.

Buhler, D.D. (2002). Challenges and opportunities for integrated weed management. Weed Science 50: 273-280.

Busse, M.D., Ratcliff, A.W., Shestak, C.J. & R.F. Powers. (2001). Glyphosate toxicity and the effects of long-term vegetation control on soil microbial communities. Soil Biol. Biochem. 33: 1777-1789.

Butler, S. J., Vickery, J. A. &Norris, K. (2007) Farmland Biodiversity and the Footprint of Agriculture. Science 315: 381-384.

BVL Bundesamt für Verbraucherschutz und Lebensmittelsicherheit (2014). EU-Bewertung von Glyphosat geht in die nächste Phase. <http://www.bvl.bund.de/DE/08_PresseInfothek/01_FuerJournalisten/01_Presse_und_Hintergrundinformationen/04_Pflanzenschutzmittel/2014/2014_01_06_pi_glyphosat.html>

Cakmak, I., Yazici, A., Tutus, Y. & Ozturk, L. (2009). Glyphosate reduced seed and leaf concentrations of calcium, manganese, magnesium, and iron in non-glyphosate resistant soybean. Europ. J. Agronomy 31: 114-119.

Catacora-Vargas, G., Galeano, P., Agapito-Tenfen, S.Z., Aranda, D., Palau, T. & Nodari, R.O. (2012). Soybean production in the Southern Cone of the Americas: Update on land and pesticide use. <http://www.genok.com/news_cms/2012/july/report-soybean-production-in-the-southern-cone-of-the-americas-update-on-land-and-pesticide-use/158>

Cathcart, R.J., Topinka, A.K., Kharbanda, P., Lange, R., Yang, R. & Hall, L.M. (2006). Rotation length, canola variety and herbicide resistance system affect weed populations and yield. Weed Science 54: 726–734.

Cerdeira, A.L. & Duke, S.O. (2006). The current status and environmental impacts of glyphosate-resistant crops: a review. J. Environm. Qual. 35: 1633–1658.

Chamberlain, D.E., Fuller, R.J., Bunce, R.G.H., Duckworth, J.C. & Shrubb, M. (2000). Changes in the abundance of farmland birds in relation to the timing of agricultural intensification in England and Wales. J Appl Ecol 37: 771-788.

Champion, G.T., May, M.J., Bennett, S, Brooks, D.R., Clark, S.J., Daniels, R.E., Firbank, L.G., Haughton, A.J., Hawes, C., Heard, M.S. et al. (2003). Crop management and agronomic context of the Farm Scale Evaluation of genetically modified herbicide tolerant crops. Philos Trans R Soc Lond B Biol Sci 358: 1801-1818.

Chapell, J.C. & LaValle, L.A. (2011). Food Security and biodiversity: can we have both? An agroecological analysis. Agric. Hum. Values. 28: 3-26.

Christoffers, M.J. & Varanasi, A.V. (2010). Glyphosate resistance: genetic basis in weeds. In: Glyphosate resistance in crops and weeds. Ed. Nandula, V.K., Wiley, New Jersey, 141-148.

Clair E, Linn L, Travert C, Amiel C, Séralini GE, Panoff JM. (2012). Effects of Roundup(®) and glyphosate on three food microorganisms: Geotrichum candidum, Lactococcus lactis subsp. cremoris and Lactobacillus delbrueckii subsp. bulgaricus. Curr Microbiol. 486-491.

Council of the European Union (2008). Council Conclusions on Genetically Modified Organisms (GMOs), Brussels, 4 December 2008. <http://www.consilium.europa.eu/ueDocs/cms_Data/docs/pressdata/en/envir/104509.pdf>

Cox, C. & Surgan, M. (2006). Unidentified inert ingredients in pesticides: Implications for humans and environmental health. Environmental Health Perspectives 114: 1803-1806.

CTB (2000). (Board for the Authorisation of Pesticides, Netherlands) Milieu-evaluatie werkzame stof: glyfosaat 8-10-99, College voor de Toelating van Bestrijdingsmiddelen, Wageningen.

Cuhra, M., Traavik, T., Dando, M., Primicerio, R., Holderbaum, D.F. & Bohn, T. (2015). Glyphosate-residues in Roundup-Ready soybean impair Daphnia magna life-cycle. J. Agric. Chem. Environ. 4: 24-36.

Czarnak-Klos, M. & Rodríguez-Cerezo, E. (2010). Best Practice Documents for coexistence of genetically modified crops with conventional and organic farming. 1. Maize crop production. European Coexistence Bureau (ECoB). <http://publications.jrc.ec.europa.eu/repository/bitstream/111111111/15705/1/jrc59319.pdf>

Dale, P.J., Clarke, B. & Fontes, E.M.G. (2002). Potential for the environmental impact of transgenic crops. Nature Biotechnol 20: 567-574.

Darmency, H. (2012). Pleiotropic effects of herbicide-resistance gene on crop yield. A review. Pest Manag Sci 69: 897-904.

Darmency, H., Klein, E.K., Gestat de Garambé, T., Gouyon, P.H., Richard-Molard, R. & Muchembled, C. (2009). Pollen dispersal in sugar beet production fields. Theor. Appl. Genet. 118: 1083-1092.

Davis, A.S., Hill, J.D., Chase, C.A.,Johanns, A.M. & Liebman, M. (2012). Increasing cropping system diversity balances productivity, profitability and environmental health. PLoS ONE 7(10): e47149. doi:10.1371/journal.pone.0047149.

De Carvalho, L.B., Rojano-Delgado, A.M., da Costa Aguiar Alves, P.L. & De Prado, R. (2013). Differential content of glyphosate and its metabolites in Digitaria insularis biotypes. Comm. Plant Sciences 3: 17-20.

de Snoo, G.R. & van der Poll, R.J. (1999). Effect of herbicide drift on adjacent boundary vegetation. Agric Ecosyst Environ 73: 1-6.

Devaux, C., Klein, E.K., Lavigne, C., Sausse, C. & Messean, A. (2008). Environmental and landscape effects on cross-pollination rates observed at long distance among French oilseed rape Brassica napus commercial fields. J. of Applied Ecology 45: 803–812.

Dewar, A.M., Haylock, L.A., Bean, K.M. & May, M.J. (2000). Deleayed control of weeds in glyphosate-tolerant sugar beet and the consequrences on aphid infestation and yield. Pest Manag Sci 56: 345-350.

Dewar, A.M., May, M.J. & Pidgeon, J.D. (2002). Management of GM herbicide-tolerant sugar beet for potential environmental benefit to farmland birds. ICAR Annual Report 2001-2002.

Dewar, A.M., May M.J., Woiwod, I.P. Haylock, L.A., Champion, G.T., Garner, B.H., Sands, R.J.N., Qi, A.M. & Pidgeon, J.D. (2003). A novel approach to the use of genetically modified herbicide tolerant crops for environmental benefit. Proc R Soc Lond B Biol Sci. 270: 335-340.

Dewar, A.M., Champion, G.T., May, M. J. & Pidgeon, J. D. (2005). The UK Farm Scale Evaluations of GM crops – A post script. Outlooks on Pest Management 16 (4): 164-73.

D’Hertefeldt, T., Jørgensen R.B. & Pettersson, L.B. (2008). Long-term persistence of GM oilseed rape in the seedbank. Biol. Lett. 23: 314-317.

Diamond, G.L. & Durkin, P.R. (1997). Effects of surfactants on the toxicity of glyphosate, with reference to RODEO. <http://www.fs.fed.us/foresthealth/pesticide/pdfs/Surfactants.pdf>

Dinelli, G., Marotti, I., Bonetti, A., Catizone, P., Urbano, J.M. & Barnes, J. 2008. Physiological and molecular bases of glyphosate resistance in Conyza canadensis biotypes from Spain. Weed Research 48: 257-265.

Dorn, E., Görlitz, G., Heusel, R. & Stumpf, K. (1992). Verhalten von Glufosinat-Ammonium in der Umwelt – Abbau im und Einfluss auf das Ökosystem. Z. PflKrankh. PflSchutz, Sonderh. XIII: 459-468.

Druille, M., Omacini, M., Golluscio, R.A. & Cabello, M.N. (2013). Arbuscular mycorrhizal fungi are directly and indirectly affected by glyphosate application. Appl. Soil Ecology 72: 143-149.

Duelli, P. (1997). Biodiversity Evaluation in Agricultural Landscapes: An Approach at two different Scales. Agric Ecosyst Environ 62: 81-91.

Duke, S.O., Wedge, D.E., Cerdeira, A.L. & Matallo, M.B. (2007). Herbicide effects on plant disease. Outlooks on Pest Management 18: 36-40.

Dun, B.Q., Wang,X.J., Lu,W., Chen,M., Zhang,W., Ping,S.Z.,et al. (2014). Development of highly glyphosate-tolerant tobacco by coexpression of glyphosateacetyltransferase gat and EPSPSG2-aroA genes. Crop J. 2, 164–169. doi:10.1016/j.cj.2014.03.003.

Durkin, P.R. (2003). Glyphosate – human health and ecological risk assessment. Final Report. Report prepared for the USDA Forest Service. <http://www.fs.fed.us/r5/hfqlg/publications/herbicide_info/2003_glyphosate.pdf>

Dzinaj, T., Kleine Hörstkamp, S., Linz, A., Ruckelshausen, A., Böttger, O., Kemper, M., Marquering, J., Naescher, J., Trautz, D. & Wigerodt, E. (1998). Multi-Sensor-System zur Unterscheidung von Nutzpflanzen und Beikräutern, Z. PflKrankh. PflSchutz, Sonderheft XVI: 233-242.

EC (2000). Economic impacts of genetically modified crops in the agri-food sector. A first review. Working Dokument Rev. 2. <http://ec.europa.eu/agriculture/publi/gmo/full_en.pdf>

EC (2002). European Commission Health & Consumer Protection Directorate-General. Directorate E – Food Safety: plant health, animal health and welfare, international questions. E1 Plant Health. 2002. Review report for the active substance glyphosate, Glyphosate 6511/IV/99-final, 21. Januar 2002, Brüssel. <http://ec.europa.eu/food/fs/sfp/ph_ps/pro/eva/existing/list1_glyphosate_en.pdf>

EC (2010). European Commission Directive 2010/77/EU of 10 November 2010 amending Council Directive 91/414/EEC as regards the expiry dates for inclusion in Annex I of certain active substances. OJ L 230, 19.8.1991.

EC (2011). European Commission Implementing Regulation (EU) No 540/2011 of 25 May 2011 implementing Regulation (EC) No 1107/2009 of the European Parliament and of the Council as regards the list of approved active substances. <http://eur-lex.europa.eu/LexUriServ/LexUriServ.do?uri=OJ:L:2011:153:0001:0186:EN:PDF>

EC (2012). EU biodiversity action plan. <http://ec.europa.eu/environment/nature/biodiversity/comm2006/index_en.htm>

Edwards C.A., Sunderland, K.D. & George, K.S. (1979). Studies on polyphagous predators of cerael aphids. J. appl. Ecol. 16: 811-823.

EFSA (2005). EFSA Scientific Report. Conclusion regarding the peer review of the pesticide risk assessment of the active substance glufosinate. 27: 1-81.

EFSA (2014). Conclusion on the peer review of the pesticide risk assessment of the active substance 2,4-D. EFSA Journal 12(9):3812. doi:10.2903/j.efsa.2014.3812.

EFSA (2015). Conclusion on the peer review of the pesticide risk assessment of the active substance glyphosate. EFSA Journal 13(11): 4302. doi: 10.2903/j.efsa.2015.4302.

Egan, J.F., Bohnenblust, E., Goslee, S., Mortensen, D. & Tooker, J. (2014).Herbicide drift can affect plant and arthropod communities. Agriculture, Ecosystems & Environment 185: 77-87.

Eker, S., Oztuk, L., Yazici, A., Erenoglu, B., Romheld, V. & Cakmak, I. (2006). Foliar-applied glyphosate substantially reduced uptake and transport of iron and manganese in sunflower (*Helianthus annuus* L.) plants. J Agric Food Chem. 54: 10019-10025.

Elmore, R.W., Roeth, F.W., Nelson, L.A., Shapiro, C.A., Klein, R.N., Knezevic, S.Z. & Martin, A. (2001). Glyphosate-Resistant Soybean Cultivar Yields Compared with Sister Lines. Agron J 93: 408-412.

Faber, M.J., Thompson, D.G., Stephenson, G.R. & Kreutzweiser, D.P. (1998). Impact of glufosinate-ammonium and bialaphos on the zooplankton community of a small eutrophic northern lake. Environ. Toxicol. Chem. 17: 1291-1299.

Fernandez, P., Gauvrit, C., Barro, F., Menendez, J. & De Prado, R. (2015). First case of glyphosate resistance in France. Agron. Sustain. Dev. DOI 10.1007/s13593-015-0322-1.

Fernandez-Cornejo J. & Caswell M. (2006). The first decade of genetically engineered crops in the United States. USDA Information Bulletin Number 11.

Fernandez-Cornjeo, J., Wechsler, S., Livingston, M. & Mitchell, L. (2014). Genetically engineered crops in the United States. Economic Research Report 162. <http://www.ers.usda.gov/media/1282246/err162.pdf>

Firbank, L.G & Forcella, F. (2000). Genetically modified crops and farmland biodiversity. Science 289 (9): 1481-1482.

Firbank, L.G., Heard, M.S., Woiwod, I.P., Hawes, C., Haughton, A.J., Champion, G.T., Scott, R.J., Hill, M.O., Dewar, A.M., Squire, G.R., et al. (2003a). An introduction to the Farm-Scale Evaluations of genetically modified herbicide-tolerant crops. J Appl Ecol 40: 2-16.

Firbank, L.G., Perry, J.N., Squire, G.R., Bohan, D.A., Brooks, D.R., Champion, G.T., Clark, S.J., Daniels, R.E., Dewar, A.M., Haughton, A.J. et al. (2003b). The implications of spring-sown genetically modified herbicide-tolerant crops for farmland biodiversity: A commentary on the Farm Scale Evaluations of Spring Sown Crops. <http://www.rothamsted.ac.uk/pie/sadie/reprints/firbank_et_al_commentary.pdf>

Firbank, L.G., Petit, S., Smart, S., Blain, A. & Fuller, R.J. (2008). Assessing the impacts of agricultural intensification on biodiversity: a British perspective. Phil. Trans. R. Soc. B 363: 777-787.

Foley, J.A., Ramankutty, N., Brauman, K.A., Cassidy, E.S., Gerber, J.S., Johnston, M., Mueller, N.D., O’Connell, C., Ray, D.K., West, P.C. et al (2011). Solutions for a cultivated planet. Nature 478: 337-342.

Fontanelli, M., Frasconia, C., Martelloni, L., Pirchio, M., Raffaelli, M. & Peruzzi, A. (2015). Innovative strategies and machines for physical weed control in organic and integrated vegetable crops. Chemical Engineering Transactions 44: 211-216.

Forlani, G., Pavan, M., Gramek, M., Kafarski, P. & Lipok, J. (2008). Biochemical bases for a widespread tolerance of cyanobacteria to the phosphonate herbicide glyphosate. Plant Cell Physiol 49: 443-456.

Fraser, K. (2013). Glyphosate Resistant Weeds – Intensifying. <http://www.stratusresearch.com/blog07.htm>

Freckleton, R.P., Stephens P.A., Sutherland W.J. & Watkinson A.R. (2004). Amelioration of biodiversity of genetically modified crops: predicting transient versus long-term effects. Proc R Soc Lond B Biol Sci 271: 325-331.

Gaines, T.A., Zhang, W., Wang, D., Bukun, B., Chisholm, S.T., Shaner, D.L., Nissen, S.J., Patzoldt, W.L., Tranel, P.J., Culpepper, A.S. et al. (2010). Gene amplification confers glyphosate resistance in Amaranthus palmeri. PNAS 107: 1029-1034.

Garibaldi, L.A., Marcelo, A.A., Klein, A.M., Cunningham, S.A. & Harder, L.D. (2011). Global growth and stability of agricultural yield decrease with pollinator dependence. PNAS 108 : 5909-5914.

Gaupp-Berghausen, M., Hofer, M., Rewald, B. & Zaller, J.G. (2015). Glyphosate-based herbicides reduce the activity and reproduction of earthworms and lead to increased soil nutrient concentrations. Sci. Rep. 5, 12886; doi: 10.1038/srep12886.

Ge, X., d'Avignon, D.A., Ackerman, J.J.H. & Sammons, R.D. (2010) Rapid vacuolar sequestration: the horseweed glyphosate resistance mechanism. Pest Manag. Sci 66: 345–348.

Ghersa, C.M., Benech-Arnold, R.I., Satorre, E.H. & Martinez-Ghersa, M.A. (2000). Advances in weed management strategies. Field Crops Res. 67: 95-104.

Gianessi, L.P. (2008). Review - Economic impacts of glyphosate-resistant crops. Pest Manag Sci 64: 346-352.

Gibbons, D.W., Bohan, D.A., Rothery, P., Stuart, R.C., Haughton, A.J., Scott, R.J., Wilson, J.D., Perry, J.N., Clark, S.J., Dawson, R.J.G. & Firbank, L.G. (2006). Weed seed resources for birds in fields with contrasting conventional and genetically modified herbicide-tolerant crops. Proc. R. Soc. B 273: 1921–1928.

Giesy, J. P., Dobson, S. & Solomon, K. R. (2000). Ecotoxicological risk assessment for Roundup herbicide, Reviews of environmental contamination and toxicology 167: 35-120, New York.

Gliddon, C.J. (1999). Gene flow and risk assessment. In: Gene Flow and Agriculture. Relevance for transgenic crops. British Crop Protection Council, Symposium Proceedings No. 72: 49-56.

Green, J.M. & Castle, L.A. (2010). Transitioning from single to multiple herbicide-resistant crops. In: Glyphosate resistance in crops and weeds. Ed. Nandula, V.K., Wiley Hoboken 67-91.

Green, J.M. (2014). Current state of herbicides in herbicide-resistant crops. Pest Manag Sci 70: 1351-1357.

Guerrero, I., Morales, M.B., Oñate, J.J., Geiger, F., Berendse, F., de Snoo, G., Eggers, S., Pärt, T., Bengtsson, J., Clement, L.W. et al. (2012). Response of ground-nesting birds to agricultural intensification across Europe: Landscape and field level management factors. Biological Conservation 152: 74-80.

Gulden, R. H., Shirtliffe, S. J. & Thomas, A. G. (2003). Harvest losses of canola (Brassica napus) cause large seedbank inputs. Weed Sci 51: 83-86.

Guo, B., Guo, Y., Hong. H., Jin, L., Zhang, L., Chang, R.Z., Lu. W., Lin, M. & Qiu L.J. (2015). Co-expression of *G2-EPSPS* and glyphosate acetyltransferase *GAT* genes conferring high tolerance to glyphosate in soybean. Frontiers in Plant Science 6: 847.

Guan, Z. J., Zhang, P. F., Wei, W., Mi, X. C., Kang, D. M., & Liu, B. (2015). Performance of hybrid progeny formed between genetically modified herbicide-tolerant soybean and its wild ancestor. AoB Plants, plv121.

Gurian-Sherman, D. (2009). Failure to yield: Evaluating the performance of genetically modified crops. <http://www.ucsusa.org/food_and_agriculture/our-failing-food-system/genetic-engineering/failure-to-yield.html>

Gustafson D.I. (2008). Sustainable use of glyphosate in North American cropping systems. Pest Manag Sci 64: 409-416.

Hall, L., K. Topinka, J. Huffman, L. Davis & Good, A. (2000). Pollen flow between herbicide-resistant Brassica napus is the cause of multiple-resistant B. napus volunteers. Weed Science 48: 688-694.

Hammond, B., Goldstein, D.A., & Saltmiras, A., (2013). Letter to the editor. Food Chem. Toxicol. 53: 459-464.

Harker, K.N. & O’Donnovan, J.T. (2013). Recent weed control, weed management, and integrated weed management. Weed Technology 27: 1-11.

Hartzler, R.G. (2010). Reduction in common milkweed (Asclepias syriaca) occurrence in Iowa cropland from 1999 to 2009. Crop Protection 29: 1542-1544.

Hassan, S. A., Bigler, F., Bogenschütz, H., Boller, E., Brun, J., Chiverton, C., Edwards, P., Mansour, F., Naton, E., Oomen, P. A. et al. (1988). Results of the fourth joint pesticide testing programme carried out by the IOBC/WPRS-Working Group Pesticides and Beneficial Organisms. J. Appl. Entomol. 105: 321-329.

Haughton, A.J., Champion, G.T., Hawes, C., Heard, M.S., Brooks, D.R., Bohan, D.A., Clark, S.J., Dewar, A.M., Firbank, L.G., Osborne, J.L. et al. (2003). Invertebrate responses to the management of genetically modified herbicide-tolerant and conventional spring crops. II. Within-field epigeal and aerial arthropods. Philos Trans R Soc Lond B Biol Sci 358: 1863-1877.

Hawes, C., Haughton, A.J., Osborne J.L., Roy, D.B., Clark, S.J., Perry, J.N., Rothery, P., Bohan, D.A., Brooks, D.R., Champion, G.T. et al. (2003). Responses of plants and invertebrate trophic groups to contrasting herbicide regimes in the Farm Scale Evaluations of genetically modified herbicide-tolerant crops. Philos Trans R Soc Lond B Biol Sci 58: 1899-1913.

Hawes, C., Squire, G.R., Hallett, P.D., Watson, C.A. & Young, M. (2010). Arable plant communities as indicators of farming practice. Agriculture, Ecosystems and Environment 138: 17-26.

Hayes, A. W. (2013a). Elsevier Announces Article Retraction from Journal Food and Chemical Toxicology. http://www.elsevier.com/about/press-releases/research-and-journals/elsevier-announces-article-retraction-from-journal-food-and-chemical-toxicology.

Hayes, A.W. (2013b). Food and Chemical Toxicology Editor-in-Chief, A. Wallace Hayes, Responds to Letters to the Editors. http://www.journals.elsevier.com/food-and-chemical-toxicology/news/editor-in-chief-a-wallace-hayes-responds-to-letters/.

Heap, I. (2016). The International Survey of Herbicide Resistant Weeds. Online February 2016. http://weedscience.org/.

Heard, M.S., Hawes, C., Champion, G.T., Clark, S.J., Firbank, L.G., Haughton, A.J., Parish, A.M., Perry, J.N., Rothery, P., Scott, R.J. et al. (2003a). Weeds in fields with contrasting conventional and genetically modified herbicide-tolerant crops. I. Effects on abundance and diversity. Philos Trans R Soc Lond B Biol Sci 358: 1819-1832.

Heard, M.S., Hawes, C., Champion, G.T., Clark, S.J., Firbank, L.G., Haughton, A.J., Parish, A.M., Perry, J.N., Rothery, P., Roy, D.B. et al. (2003b). Weeds in fields with contrasting conventional and genetically modified herbicide-tolerant crops. II. Effects on individual species. Philos Trans R Soc Lond B Biol Sci 358: 1833-1846.

Heard, M.S., Rothery, P., Perry, J.N. & Firbank, L.G. (2005). Predicting longer-term changes in weed populations under GMHT management. Weed Res 45: 331-338.

Heinemann, J.A., Massaro, M., Coray, D.S., Zanon Agapito-Tenfen, S. & Dale Wen, J. (2014). Sustainability and innovation in staple crop production in the US Midwest. Int. J. of Agricultural Sustainability 12: 71-88.

Herbert, L.T., Vázquez, D.E., Arenas, A. & Farina, W.M. (2014). Effects of field-realistic doses of glyphosate on honeybee appetitive behavior. J. Experimental Biology 217: 3457-3464.

Heydemann, B. (1983). Aufbau von Ökosystemen im Agrarbereich und ihre langfristigen Veränderungen, Daten und Dokumente zum Umweltschutz, Sonderreihe Umwelttagung 35: 53-84.

Hilbeck, A., Meier, M. & Benzler, A. (2008). Identifying indicator species for post-release monitoring of genetically modified, herbicide resistant crops. Euphytica 164: 903-912.

Holt, E., Weber, R., Stevenson, G. & Gaus, C. (2010). Polychlorinated dibenzo-p-dioxins and dibenzofurans (PCDD/Fs) impurities in pesticides: A neglected source of contemporary relevance. Environ. Sci. Technol. 44: 5409–5415.

Holtzapffel, R., Mewett, O., Wesley, V. & Hattersley, P. (2008). Genetically modified crops: tools for insect pest and weed control in cotton and canola, Australian Government Bureau of Rural Sciences, Canberra. Published: November 2008 pp. 99.

Hüsken, A. & Dietz-Pfeilstetter, A. (2007). Pollen-mediated intraspecific gene flow from herbicide resistant oilseed rape (Brassica napus L.). Transgenic Res. 16: 557-569.

IAASTD (2009). Agriculture at a crossroads. International Assessment of Agricultural Knowledge, Science and Technology for Development. <http://www.unep.org>

IARC International Agency for Research on Cancer (2015). Monograph on Glyphosate. <http://monographs.iarc.fr/ENG/Monographs/vol112/mono112-02.pdf>

Jackson, R.E. & Pitre, H.N. (2004). Influence of Roundup Ready soybean production systems and glyphosate application on pest and beneficial insects in narrow-row soybean. J. Entomol Sci 39: 62-70.

James, C. (2013). Global status of commercialized biotech/GM crops: 2013. ISAAA Briefs 46. ISAAA. Ithaca, NY, USA. [www.isaaa.org](http://www.isaaa.org)

Jansen, C., Schuphan, I. & Schmidt, B. (2000). Glufosinate metabolism in excised shoots and leaves of twenty plant species. Weed Sci 48: 319-326.

Jasieniuk, M. (1995). Constraints on the evolution of glyphosate resistance in weeds. Resistant Pest Management Newsletter 7: 31-32.

Jasinski, J., Eisley, B., Young, C., Willson, H. & Kovach, J. (2004). Beneficial Arthropod Survey in Transgenic and Non-Transgenic Field Crops in Ohio. <http://ohioline.osu.edu/sc179/sc179_34.html>

Jørgensen, R.B., Hauser, T., D’Hertefeldt, T., Andersen, N.S, & Hooftman, D. (2009). The variability of processes involved in transgene dispersal – case study from Brassica and related genera. Environ. Sci. Pollut. Res. 16: 389-395.

Johal, G.R. & Huber, D.M. (2009). Glyphosate effects on diseases of plants. Eur J Agron 31: 144-152.

Johnson, W.G., Davis, V.M., Kruger, G.R. & Weller, S.C. (2009). Influence of glyphosate-resistant cropping systems on weed species shifts and glyphosate-resistant weed populations. Europ. J. Agronomy 31: 162-172.

Johnson, W.G., Hallett, S.G., Legleiter, T.R., Whitford, F., Weller, S.C., Bordelon, B.P. & Lerner, B.R. (2012). 2,4-D- and dicamba-tolerant crops - some facts to consider. [www.extension.purdue.edu](http://www.extension.purdue.edu)

Jones, D.K., Hammond, J.I. & Relyea, R.A. (2011). Competitive stress can make the herbicide Roundup® more deadly to larval amphibians. Environ. Toxicol. Chem. 30: 446-454.

Kalaitzandonakes, N.G. & Suntornpithug, P. (2001). Why do farmers adopt biotech cotton? Proceedings of the Beltwide Cotton Conference 1: 179-183, National Cotton Council, Memphis TN.

Kaundun, S.S., Zelaya, I.A., Dale, R.P., Lycett, A.J., Carter, P., Sharples, K.R. & McIndoe, E. (2008). Importance of the P106S target-site mutation in conferring resistance to glyphosate in a goosegrass (Eleusine indica) population from the Philippines. Weed Science 55: 637-646.

Kawata, M., Murakami, K. & Ishikawa, T. (2009). Dispersal and persistence of genetically modified oilseed rape around Japanese harbors. Env. Science Pollution Res. 16: 120-126.

Kelly, D.W., Poulin, R., Tompkins, D.M. & Townsend, C.R. (2010): Synergistic effects of glyphosate formulation and parasite infection on fish malformations and survival. J Appl Ecol 47: 498–504.

Khan, M. F. (2015). Update on Adoption of Glyphosate-Tolerant Sugar Beet in the United States. Outlooks on Pest Management, 26: 61-65.

King, C.A., Purcell, L.C. & Vories, E.D. (2001). Plant Growth and Nitrogenase Activity of Glyphosate-Tolerant Soybean in Resonse to Foliar Glyphosate Applications. Agron J 93: 179-186.

King, C. A., & Purcell, L.C. ( 2001). Soybean nodule size and relationship to nitrogen fixation response to water deficit. Crop Sci 41: 1099–1107.

Kleter, G.A., Harris, C., Stephenson, G. & Unsworth, J. (2008). Comparison of herbicide regimes and the associated potential environmental effects of glyphosate-resistant crops versus what they replace in Europe. Pest Manag Sci 64: 479-488.

Knispel, A.L. & McLachlan, S.M. (2009). Landscape-scale distribution and persistence of genetically modified oilseed rape (Brassica napus) in Manitoba, Canada. Environ Sci Pollut R 2009 Jul 9. [Epub ahead of print].

Knispel, A.L., McLachlan, S.M., Van Acker, R.C., Lyle F. & Friesen, L.F. (2008). Gene flow and multiple herbicide resistance in escaped canola populations. Weed Science 56: 72-80.

Korr, V., Maidl, F.-X. & Fischbeck, G. (1996). Auswirkungen direkter und indirekter Regulierungsmaßnahmen auf die Unkrautflora in Kartoffeln und Weizen. Z. PflKrankh. PflSchutz, Sonderheft XV: 349-358.

Kortekamp, A. (2011). Unexpected side effects of herbicides: modulation of plant-pathogen interactions. In: Herbicides and Environment. www.intechopen.com/download/pdf/12583‎.

Krebs, J. R., Wilson, J. D., Bradbury, R. B. & Siriwardena, G.M. (1999). The second Silent Spring? Nature 400: 611-612.

Kremer, R.J., Means, N.E. & Kim, S. (2005). Glyphosate affects soybean root exudation and rhizosphere microorganisms. Int. J. of Analytical Environmental Chemistry 85: 1165-1174.

Kremer, R.J. & Means, N.E. (2009). Glyphosate and glyphosate-resistant crop interactions with rhizosphere microorganisms. Eur J Agron 31: 153–161.

Kromp, B. (1999). Carabid beetles in sustainable agriculture: a review on pest efficiacy, cultivation impacts and enhancement. Agric Ecosyst Environ 74: 187-228.

Kurenbach, B., Marjoshi, D., Amábile-Cuevas, C.F., Ferguson, G.C., Godsoe, W., Gibson, P., Heinemann, J.A. (2015). Sub-lethal exposure to commercial formulations of the herbicides dicamba, 2,4-dichlorophenoxyacetic acid, and glyphosate cause changes in antibiotic susceptibility in *Escherichia coli* and *Salmonella enterica* serovar Typhimurium. mBio 6(2):e00009-15. doi:10.1128/mBio.00009-15.

Kutlesa, N.J. & Caveney, S. (2001). Insecticidal activity of glufosinate through glutamine depletion in a caterpillar. Pest Manag. Sci. 57: 25-32.

Leech, D. (2002). Factors affecting the survival of Birds of Conservation Concern. Technology 20: 485-493.

Legleiter, T.R. & Bradley, K.W. (2008). Glyphosate and multiple herbicide resistance in common waterhemp (Amaranthus rudis) populations from Missouri. Weed Science 55: 582-587.

Lettner, J., Hank, K. & Wagner, P. (2001). Ökonomische Potenziale der teilflächenspezifischen Unkrautbekämpfung. BerLdw. 79: 107-137.

Lingenfelter, D. & Curran, W. (2013). What’s new for agronomic weed control in 2013? http://extension.psu.edu/plants/crops/news/2013/01/herbicide-resistant-crops.

Loening, U.E. (2015). A challenge to scientific integrity: a critique of the critics of the GMO rat study conducted by Gilles-Eric Séralini et al. (2012). Environmental Sciences Europe Vol 27:13.

Loomis, D., Guyton, K., Grosse, Y., El Ghissassi, F., Bouvard, V., Benbrahim-Tallaa, L., Guha, N., Mattock, H. & Straif, K. (2015). Carcinogenicity of lindane, DDT, and 2,4-dichlorophenoxyacetic acid. Lancet Oncol. 16: 891-892.

Lu, B.-R. (2005). Multidirectional gene flow among wild, weedy, and cultivated soybean. In Gressel J. (ed) Crop Ferality and Volunteerism. Taylor and Francis: 137-147.

Lu, B.-R. & Snow, A. (2005). Gene flow from genetically modified rice and its environmental consequences. Bioscience 55: 669-678.

Lundgren, J.G. & Fausti, S.W. (2015). Trading biodiversity for pest problems. Sci Adv. 1, e1500558.

Malatesta, M., Perdoni, F., Santin, G., Battistelli, S., Muller, S., & Biggiogera, M. (2008). Hepatoma tissue culture (HTC) cells as a model for investigating the effects of low concentrations of herbicide on cell structure and function. Toxicol in vitro 22: 1853-1860.

Mallory-Smith, C. & Zapiola, M. (2008). Gene flow from glyphosate-resistant crops. Pest Manag Sci 64: 428–440.

Mann, R.M., Bidwell, J.R. & Tyler, M.J. (2003). Toxicity of herbicide formulations to frogs and the implications for product registration: A case study from Western Australia. Appl Herpetology 1: 13-22.

Marshall, E.J.P., Brown, V.K., Boatman, N.D., Lutman, P.J.W., Squire, G.R. & Ward, L.K. (2003). The role of weeds in supporting biological diversity within crop fields. Weed Research 43: 77-89.

Mauro, I. & McLachlan, S.M. (2003). Risk analysis of genetically modified crops on the canadian prairies. In: Technical Workshop on the Management of Herbicide Tolerant (HT) Crops. CFIA, Plant Products Directorate, Plant Biosafety Office (Ed.). Ottawa, Ontario, September 9-10, 2003: 12-13.

McNaughton, K.E., Letarte, J., Lee, E.A., & Tardif, F.J. (2005). Mutations in ALS confer herbicide resistance in redroot pigweed (Amaranthus retroflexus) and Powell amaranth (Amaranthus powellii). Weed Sci 53: 17-22.

Means, N.E., Kremer, R.J. & Ramsier, C. (2007). Effects of glyphosate and foliar amendments on activity of microorganisms in the soybean rhizosphere. J Environ Sci Heal B 42: 125-32.

Mesnage, R., Bernay, B. & Séralini, G.E. (2013). Ethoxylated adjuvants of glyphosate-based herbicides are active principles of human cell toxicity. Toxicology 131: 122-8.

Mesnage, R., Arno, M., Costanzo, M., Malatesta, M., Séralini, G.E. & Antoniou, M.N. (2015). Transcriptome profile analysis reflects rat liver and kidney damage following chronic ultra-low dose Roundup exposure. Environmental Health 14: 70.

Meyer, H. & Hilbeck, A. (2013). Rat feeding studies with genetically modified maize - a comparative evaluation of applied methods and risk assessment standards. Environmental Sciences Europe 2013, 25:33 doi:10.1186/2190-4715-25-33.

Mithila, J., Swanton, C.J., Blackshaw, R.E., Cathcart, R.J. & Hall, J.C. (2008). Physiological basis for reduced glyphosate efficacy on weeds grown under low soil nitrogen. Weed Science 56: 12-17.

Monroy, C.M., Cortes, A.C., Sicard, D.M. & de Restrepo, H.G. (2005). Cytotoxicity and genotoxicity of human cells exposed in vitro to glyphosate. Biomedica 25: 335-345.

Monsanto Canada, Inc. (2002). Material Safety Data Sheet, Roundup Original. [www.mta.ca](http://www.mta.ca)

Morandin, L.A. & Winston, M.L. (2005). Wild bee abundance and seed production in conventional, organic and genetically modified canola. Ecological Applications 15: 871-881.

Morjan, W.E. & Pedigo, L.P. (2002). Suitability of transgenic glyphosate-resistant soybean to green cloverworm (Lepidoptera: Noctuidae). J Econ Entomol 95: 1275-1280.

Morjan, W.E., Pedigo, L.P. & Lewis, L.C. (2002). Fungicidal effects of glyphosate and glyphosate formulations on four species of entomopathogenic fungi. Environ. Entomology 31: 1206-1212.

Mortensen, D.A., Egan, J.F., Maxwellm, B.D., Ryan, M.R., & Smith, R.G. (2012). Navigating a critical juncture for sustainable weed management. Biosci. 62:75–85.

Mulik (2015). Economic Impacts of Diversified Cropping Systems. Selected Paper prepared for presentation at the 2015 Agricultural & Applied Economics Association and Western Agricultural Economics Association Annual Meeting, San Francisco, CA, July 26-28. <http://ageconsearch.umn.edu/bitstream/205805/2/AAEA%202015%20paper.pdf>

Myers, J.P., Antoniou, M.N., Blumberg, B., Carroll, L., Colborn, T., Everett, L.G., Hansen, M., Landrigan, P.J., Lanphear, B.P., Mesnage, R., Vandenberg, L.N., vom Saal, F.S., Welshons, W.V., Benbrook, C.M. et al. (2016). Concerns over use of glyphosate-based herbicides and risks associated with exposures: a consensus statement. Environmental Health 15(1): 1-13; DOI: 10.1186/s12940-016-0117-0.

Nandula, V.K., Wright, A.A., Molin, W.T., Ray, J.D., Bond, J.A. & Eubank, T.W. (2014). EPSPS amplification in glyphosate-resistant spiny amaranth (Amaranthus spinosus): A case of gene transfer via interspecific hybridization from glyphosate-resistant Palmer amaranth (Amaranthus palmeri). Pest Manag Sci DOI: 10.1002/ps.3754.

Nelson, G.C. & Bullock, D.S. (2003). Environmental effects of glyphosate resistant soybean in the United States. In: The Economic and Environmental Impacts of Agbiotech. A Global Perspective. Kalaitzandonakes, N. (ed), New York, 89-101.

Neve, P., Diggle, A.J., Smith, F.P. & Powles, S.B. (2003). Simulating evolution of glyphosate resistance in *Lolium rigidum* I: population biology of a rare resistance trait. Weed Res 43: 404-417.

Neve, P. (2007). Challenges for herbicide resistance evolution and management: 50 years after Harper. Weed Research 47: 365-369.

Nicholls, C.I. & Altieri, M.A. (2013). Plant biodiversity enhances bees and other insect pollinators in agroecosystems. Agron. Sustain. Dev. 33: 257-274.

Nordby, D, Hartzler, B. & Bradley, K. (2007). Biology and Management of Waterhemp. <http://www.glyphosateweedscrops.org/Pubs./html>

Norsworthy, J.K., Griffith, G.M., Scott, R.C., Smith, K.L. & Oliver, L.R. (2008). Confirmation and control of glyphosate-resistant palmer amaranth (*Amaranthus palmeri*) in Arkansas. Weed Tech 22: 108-113.

Norsworthy, J.K., Ward, S.M., Shae, D.R., Llewellyn, R.S., Nochols, R.L., Webster, T.M., Bradley, K.W., Frisvold, G., Powles, S.B., Burgos, N.R. et al. (2012). Reducing the risks of herbicide resistance. Best management practices and recommendations. Weed Science 60, Special Issue: 31-62.

OECD (Organisation for Economic Co-operation and Development) (1999a): Consensus Document on General Information Concerning the Genes and Their Enzymes that Confer Tolerance to Glyphosate Herbicide. Series on Harmonization of Regulatory Oversight in Biotechnology No. 10.

OECD (Organisation for Economic Co-operation and Development) (1999b): Consensus Document on General Information Concerning the Genes and Their Enzymes that Confer Tolerance to Phosphinothricin Herbicid. Series on Harmonization of Regulatory Oversight in Biotechnology No. 11.

Ormerod, S.J. & Watkinson, A.R. (2000). Editors’ Introduction: Birds and Agriculture. J Appl Ecol 37: 699-705.

Orson, J. (2002). Gene stacking in herbicide tolerant oilseed rape: lessons from the North American expierience. English Nature Research Reports 443: 17, Peterbourough.

Paganelli, A., Gnazzo, V., Acosta, H., López, S.L. &Carrasco, A.E. (2010). Glyphosate-Based Herbicides Produce Teratogenic Effects on Vertebrates by Impairing Retinoic Acid Signaling. Chem. Res. Toxicol. 23: 1586-1595.

Pedersen, B.P., Neve, P., Andreasen, Ch. & Powles, S.B. (2007). Ecological fitness of a glyphosate-resistant *Lolium rigidum* population: Growth and seed production along a competition gradient. Basic Appl Ecol 8: 258-268.

Pengue, W. A. (2004). Environmental and socio economic impacts of transgenic crops in Argentina and South America: An ecological economics approach. Naturschutz und Biologische Vielfalt 1: 49-59.

Pérez, G.L., Torremorell, A., Mugni, H., Rodriguez, P. Solange Vera, M., Do Nascimento, M., Allende, L., Bustingorry, J., Escaray, R., Ferraro, M. et al. (2007). Effects of the herbicide Roundup on freshwater microbial communities: a mesocosm study. Ecological Applications 17: 2310-2322.

Perez-Jones, A. & Mallory-Smith, C. (2010). Biochemical mechanisms and molecular basis of evolved glyphosate resistance in weed species. In: Glyphosate resistance in crops and weeds. Ed. Nandula, V.K., Wiley, New Jersey, 119-140.

Phillips, P. (2003). The economic impact of herbicide tolerant canola in Canada. In: The Economic and Environmental Impacts of Agbiotech. A Global Perspective. Kalaitzandonakes, N. (ed), New York, 119-139.

Phipps, R.H. & Park, J.R. (2002). Environmental benefits of genetically modified crops: Global and European perspectives on their ability to reduce pesticide use. J AnimFeed Sci 11: 1-18.

Pidgeon, J.D., May, M.J., Perry, J.N. & Poppy, G.M. (2007). Mitigation of indirect environmental effects of GM crops. Proc R Soc Lond B Biol Sci 274: 1475-1479.

Pivard, S., Adamczyk, K., Lecomte, J., Lavigne, C., Bouvier, A., Deville, A., Gouyon, P. H. & Huet, S. (2008). Where do the feral oilseed rape populations come from? A large-scale study of their possible origin in a farmland area. J Appl Ecol 45: 476 – 485.

Pleasants, J.M. & Oberhauser, K.S. (2013). Milkweed loss in agricultural fields because of herbicide use: effect on the monarch butterfly population. Insect Conservation and Diversity 6: 135–144.

Pleasants, J.M., Williams, E.H., Brower, L.P., Oberhauser, K.S., Taylor, O.R. (2016). Conclusion of no decline in summer monarch population not supported. Letter to the editor. Annals Entomological Society of America Doi: 10.1093/aesa/sav115.

Plötner, J. & Matschke, J. (2012). Akut-toxische, subletale und indirekte Wirkungen von Glyphosat und glyphosathaltigen Herbiziden auf Amphibien – eine Übersicht. Zeitschrift f. Feldherpetologie 19: 1-20.

Powell, J.R., Gulden, R.H., Hart, M.M., Campbell, R.G., Levy-Booth, D.J., Dunfield, K.E., Pauls, K.P., Swanton, C.J., Trevors, J., & Klironomos, J.N. (2007). Mycorrhizal and rhizobial colonization of genetically modified and conventional soybean. Appl and Environ Microbiol 73: 4365-4367.

Powell, J.R. & Swanton, C.J. (2008). A critique of studies evaluation glyphosate effects on diseases associated with Fusarium spp. Weed Research 48: 307-318.

Power, A.G. (2010). Ecosystem services and agriculture: tradeoffs and synergies. Phil. Trans. R. Soc. B 365: 2959–2971.

Powles, S.B. (2008). Evolved glyphosate-resisant weeds around the world: lessons to be learnt. Pest Manag Sci 64: 360-365.

Pratley, J., Urwin, N., Stanton, R., Baines, P., Broster, J., Cullis, K., Schafer, D., Bohn, J. & Krueger, R. (1999). Resistance to glyphosate in *Lolium rigidum*. I. Bioevaluation. Weed Sci 47: 405-411.

Preston, B.L. (2002). Indirect effects on aquatic ecotoxicology: implications for ecological risk assessment. Environ. Management 29: 311-323.

Preston, C. & Wakelin, A.W. 2008. Resistance to glyphosate from altered herbicide translocation patterns. Pest Manag Sci 64: 372-376.

Prince, J.M., Shaw, D.R., Givens, W.A., Owen, M.D.K., Weller, S.C., Young, B.G., Wilson, R.G. & Jordan, D.L. (2012a). Benchmark study: I. Introduction, weed population, and management trends form the benchmark survey 2010. Weed Technology 26: 525-530.

Prince, J.M., Shaw, D.R., Givens, W.A., Newman, M.E., Owen, M.D.K., Weller, S.C., Young, B.G., Wilson, R.G. & Jordan, D.L. (2012b). Benchmark study: II. A 2010 survey to assess grower awareness of and attitudes toward glyphosate resistance. Weed Technology 26: 531-535.

Prince, J.M., Shaw, D.R., Givens, W.A., Newman, M.E., Owen, M.D.K., Weller, S.C., Young, B.G., Wilson, R.G. & Jordan, D.L. (2012c). Benchmark study: III. Survey on changing herbicide use patterns in glyphosate-resistant cropping systems. Weed Technology 26: 536-542.

Prince, J.M., Shaw, D.R., Givens, W.A., Newman, M.E., Owen, M.D.K., Weller, S.C., Young, B.G., Wilson, R.G. & Jordan, D.L. (2012d). Benchmark study: IV. Survey of grower practices for managing glyphosate-resistant weed populations. Weed Technology 26: 543-548.

Qaim, M. & Traxler, G. (2005): Roundup Ready soybean in Argentina: farm level and aggregated welfare effects. Agr Econ 32: 73-86.

Quist, D.A., Heinemann, J.A., Myhr, A.I., Aslaksen, J. & Funtowicz, S. (2013). Hungry for innovation: pathways from GM crops to agroecology. In: Late lessons from early warnings: science, precaution, innovation. European Environment Agency Copenhagen, 458-485.

Ramsay, G., Thompson, C. & Squire, G. (2003). Quantifying Landscape-Scale Gene Flow in Oilseed Rape. Defra Project RG0216 Final Report. Department for Environment, Food and Rural Affairs, London, UK.

Reddy, K.N. & Norsworthy, J.K. (2010). Glyphosate-resistant crop production systems: Impact on weed species shifts. In: Glyphosate resistance in crops and weeds. Ed. Nandula, V.K., Wiley, New Jersey, 165-184.

Relyea, R.A. (2005). The lethal impact of Roundup® and predatory stress on six species of North American tadpoles. – Arch. Environ. Con. Tox. 48: 351-357.

Relyea, R. & Hoverman, J. (2006). Assessing the ecology in ecotoxicology: a review and synthesis in freshwater systems. Ecology Letters 9: 1157-1171.

Rieger, M.A., Lamond, M., Preston, C., Powles, S.B. & Roush, R.T. (2002). Pollen-mediated movement of herbicide resistance between commercial canola fields. Science 296, 2386-2388.

Robinson, R.A. & Sutherland, W.J. (2002). Post-war changes in arable farming and biodiversity in Great Britain. J Appl Ecol l 39: 157-176.

Rockström, J., Steffen, W., Noone, K., Persson, Å. Chapin, S., Lambin, E.F., Lenton, T.M. Scheffer, M., Folke1, M., Schellnhuber, H.J. et al. (2009). A safe operating space for humanity. Nature 461: 472-475.

Romero, A., Chamorro, L. & Sans, F.X. (2008). Weed diversity in crop edges and inner fields of organic and conventional dryland winter cereal crops in NE Spain. Agric. Ecosyst. Envir. 124: 97-104.

Roslycky, E.B. (1982). Glyphosate and the response of the soil microbiota. Soil Biology and Biochemistry 14: 87-92.

Roy, D.B., Bohan, D.A., Haughton, A.J., Hill, M.O., Osborne, J.L., Clark, S.J., Perry, J.N., Rothery, P., Scott, R.J., Brooks, D.R. et al. (2003). Invertebrates and vegetation of field margins adjacent to crops subject to contrasting herbicide regimes in the Farm Scale Evaluations of genetically modified herbicide-tolerant crops. Philos Trans R Soc Lond B Biol Sci 358: 1879-1898.

Sammons, R.D. & Gaines, T.A. (2014). Glyphosate resistance: State of knowledge. Pest Manag Sci Doi: 10.1002/ps.3743.s

Sanchis, J., Kantiani, L., Llorca, M., Rubio, F., Ginebreda, A., Fraile, J., Garrido, T. & Farré, M. (2011). Determination of glyphosate in groundwater samples using an ultrasensitive immunoassay and confirmation by on-line solid-phase extraction followed by liquid chromatography coupled to tandem mass spectrometry. Anal Bioanal Chem. 402: 2335-45.

Sanders, T. & Lassen, S. (2015). The herbicide glyphosate affects nitrification in the Elbe estuary, Germany. Geophysical Res. Abstracts 17: EGU2015-13076.

Sankula, S., Marmon, G. & Blumenthal, E. (2005). Biotechnology-Derived Crops Planted in 2004 - Impacts on US Agriculture. National Center for Food and Agricultural Policy. <http://www.ncfap.org>

Sanyal, D. & Shrestha, A. (2008). Direct effect of herbicides on plant pathogens and disease development in various cropping systems. Weed Science 56: 155-160.

Sanyal, D., Bhowmik, P.C., Anderson, R.L. & Shrestha, A. (2008). Revisiting the perspective and progress of integrated weed management. Weed Science 56: 161-167.

Schafer, M.G., Ross, A.A., Londo, J.P., Burdick, C.A., Lee, E.H., Travers, S.E., Van de Water, P.K. & Sagers, C.L. (2011). The establishment of genetically engineered canola populations in the US. PLoS ONE 6(10): e25736. doi:10.1371/journal.pone 0025736.

Schafer, J.R., Hallett, S.G. & Johnson, W.G. (2012). Response of giant ragweed (*Ambrosia trifida*), horseweed (*Conyza canadensis*), and common lambsquarters (*Chenopodium album*) biotypes to glyphosate in the presence and absence of soil microorganisms. Weed Science 60: 641-649.

Schmitz, J., Schäfer, K., Brühl, C.A. (2013). Agrochemicals in field margins – Assessing the impacts of herbicides, insecticides, and fertilizer on the common Buttercup (*Ranunculus acris*). Environ. Toxicol. Chem. 32: 1124–1131.

Schmitz, J., Schäfer, K., Brühl, C.A. (2014a). Agrochemicals in field margins – Field evaluation of plant reproduction effects. Agric. Ecosyst. Environ. 189: 82–91.

Schmitz, J., Schäfer, K., Brühl, C.A. (2014b). Agrochemicals in field margins – An experimental field study to assess the impacts of pesticides and fertilizers on a natural plant community. Agric. Ecosyst. Environ. 193: 60–69.

Schnepf, R. (2003). Genetically engineered soybeans: Acceptance and intellectual property rights issues in South America. CRS Report for Congress. [www.ipmall.info/hosted_resources/crs/RS21558_031017.pdf](http://www.ipmall.info/hosted_resources/crs/RS21558_031017.pdf)

Schoenenberger, N. & D’Andrea, L. (2012). Surveying the occurrence of subspontaneous glyphosate-tolerant genetically engineered Brassica napus L. (Brassicaeae) along Swiss railways. Env. Sciences Europe 24: 23. Doi:10.1186/2190-4715-24-23.

Schütte, G (2002). Prospects of biodiversity in herbicide-resistant crops. Outlook on Agriculture. 31: 193-198.

Schütte G. (2003). Herbicide resistance: Promises and prospects of biodiversity for European Agriculture. Agric. Hum. Values. 20: 217-230.

Schütte G. (2005). Integrated Pest Management and Genetically Engineered Plants. <http://www.uni-hamburg.de/fachbereiche-einrichtungen/biogum/publikationen_/schuette_2005_IPM_GG.pdf>

Schütte, G., Stachow, U. & Werner, A. (2004). Agronomic and Environmental Aspects of the Cultivation of Transgenic Herbicide Resistant Plants. UBA Texte 11/04, Umweltbundesamt Berlin, pp 111.

Schulze, J., Frauenknecht, T., Brodmann, P. & Bagutti, C. (2014). Unexpected diversity of feral genetically modified oilseed rape (*Brassica napus* L.) despite a cultivation and import ban in Switzerland. PLOS ONE | DOI:10.1371/journal.pone.0114477.

Séralini, G.-E., Clair, E., Mesnage, R., Gress, S., Defarge, N., Malatesta, M., Hennequin, D. & de Vendômois, J.S. (2012). Long term toxicity of a Roundup herbicide and a Roundup-tolerant genetically modified maize. Food Chem. Toxicol. 50: 4221-4231.

Séralini, G.-E., Mesnage, R., Defarge, N., Gress, S., Hennequin, D., Clair, E., Malatesta, M., & de Vendômois, J.S. (2013). Answers to critics: Why there is a long term toxicity due to a Roundup- tolerant genetically modified maize and to a Roundup herbicide. Food Chem. Toxicol. 53: 476-483.

Séralini, G.-E., Clair, E., Mesnage, R., Gress, S., Defarge, N., Malatesta, M., Hennequin, D. & de Vendômois, J.S. (2014). Republished study: long-term toxicity of a Roundup herbicide and a Roundup-tolerant genetically modified maize. Environmental Sciences Europe 26: 14. http://www.enveurope.com/content/26/1/14

Service, R.F. (2013). What happens when weed killers stop killing? Science 341: 1329.

Shaner, D.L. (2000). The impact of glyphosate-tolerant crops on the use of other herbicides and on resistance management. Pest Manag. Sci. 56: 320-326.

Shaner, D.L. (2009). Role of translocation as a mechanism of resistance to glyphosate. Weed Science 57: 118-123.

Shaw, M.W., Harwood, T.D., Wilkinson, M.J. & Elliott, L. (2006). Assembling spatially explicit landscape models of pollen and spore dispersal by wind for risk assessment. Proc. R. Soc. B 273: 1705-1713.

Shehata, A.A., Schrödl, W., Aldin, A.A., Hafez, H.M. & Krüger, M. (2013). The effect of glyphosate on potential pathogens and beneficial members of poultry micro-biota in vitro. Curr Microbiol. 66: 350-358.

Simarmata, M. & Penner, D. (2008). The basis for glyphosate resistance in rigid ryegrass (*Lolium rigidum*) from California. Weed Science 55: 181-188.

Skeff, W., Neumann, C. & Schulz-Bull, D.E. (2015). Glyphosate and AMPA in the estuaries of the Baltic Sea method optimization and field study. Marine Pollution Bulletin http://dx.doi.org/10.1016/j.marpolbul.2015.08.015.

Springett, J.A. & Gray, R.A.J. (1992). Effect of repeated low doses of biocides on the earthworm Aporrectodea caliginosa in laboratory culture. Soil Biology & Biochemistry 24: 1739-1744.

Squire, G.R., Brooks, D.R., Bohan, D.A., Champion, G.T., Daniels, R.E., Haughton, A.J., Hawes,C., Heard, M.S., Hill, M.O., May, M.J. et al. (2003). On the rationale and interpretation of the Farm Scale Evaluations of genetically modified herbicide-tolerant crops. Philos Trans R Soc Lond B Biol Sci 358: 1779-1799.

Stinner, BR. & House, G.J. (1990). Arthropods and other invertebrates in conservation-tillage agriculture. Annu Rev Entomol 35: 299-318.

Strandberg, B. & Pedersen, M.B. (2002). Biodiversity in Roundup tolerant fodder beet fields. NERI Technical Report No. 410. http://www2.dmu.dk/1_viden/2_Publikationer/ 3_fagrapporter/rapporter/FR410.pdf

Sutherland, W.J., Armstrong-Brown, S., Armsworth, P.R., Brereton, T., Brickland, J., Campbell, C.D., Chamberlain, D.E., Cooke, A.J., Dulvy, N.K., Dusic, N.R. et al. (2006) The identification of 100 ecological questions of high policy relevance in the UK. J Appl Ecol 43: 617-627.

Swanson L., Leu, A. & Abrahamson J. (2014). Genetically engineered crops, glyphosate and the deteroration of health in the United Stated of America. Journal of organic systems. 9: 6-37.

Swanton, C.J., Clements, D.R. & Derksen, A. (1993). Weed Succession under Conservation Tillage: A Hierarchical Framework for Research and Management. Weed Technol 7: 286-297.

Sweet, J., Simpson, E., Law, J., Lutman, P. J.W., Berry, K. J., Payne, R.W., Champion, G. T., May, M. J., Walker, K., Wightman, P. et al. (2004). Botanical and rotational implications of genetically modified herbicide tolerance in winter oilseed rape and sugar beet (BRIGHT Project) (H-GCA) Project Report No 353). London, Home-Grown Cereals Authority: pp. 242.

Tan, S., R. Evans, M. Dahmer, Singh, B. & Shaner, D.L. (2005). Imidazolinone-tolerant crops: History, current status, and future. Pest Manag Sci 61: 246-257.

Tappeser B., Reichenbecher W., Teichmann H. (2014). Agronomic and environmental aspects of the cultivation of genetically modified herbicide-resistant plants. A BfN-FOEN-EAA-Joint paper. BfN-Skripten 362, <http://www.bfn.de/fileadmin/MDB/documents/service/skript362.pdf>

TEEB (2008). The economics of ecosystems and biodiversity – An interim report. <http://www.teebweb.org/teeb-study-and-reports/additional-reports/interim-report/>

Thelen, K.D. & Penner, D. (2007). Yield environment affects glyphosate-resistant hybrid response to glyphosate. Crop Science 47: 2098-2107.

Thies, C., Haenke, S., Scherber, C., Bengtsson, J., Bommarco, R., Clement, L.W., Ceryngier, P., Dennis, C., Emmerson, M., Gagic, V. et al. (2011). The relationship between agricultural intensification and biological control: experimental tests across Europe. Ecological Applications 21: 2187–2196.

Thöle, H. & Dietz-Pfeilstetter, A. (2012) Molecular marker-based identification of oilseed rape volunteers with different secondary dormancy levels in oilseed rape fields. Europ. J. Agronomy 43: 194-200.

Toy, A.D.F. & E.H. Uhing (1964). Aminomethylenephosphinic acids, salts thereof, and process for their production. United States Patent Office 3,160,632. <http://www.archpatent.com/patents/3160632>

Tscharntke, T., Klein, A.M., Kruess, A., Steffan-Dewenter, I. & Thies, C. (2005). Landscape perspectives on agricultural intensification and biodiversity – ecosystem service management. Ecology Letters 8: 857-874.

Tsui, M.T. & Chu, L.M. (2004). Comparative toxicity of glyphosate-based herbicides: aqueous and sediment porewater exposure. Arch Environ Contam Toxicol 46: 316-323.

Tuck, S.L., Winqvist, C., Mota, F., Ahnström, J., Turnbull, L.A. & Bengtsson, J. (2014). Land-use intensity and the effects of organic farming on biodiversity: a hierarchical meta-analysis. J. Appl. Ecol. Doi: 10.1111/1365-2664.12219.

Tush, D. & Meyer, M.T. (2016). Polyoxyethylene tallow amine, a glyphosate formulation adjuvant: soil adsorption chracteristics, degradation profile, and occurrence on selected soils from agricultural fields in Iowa, Illinois, Indiana, Kansas, Mississippi, and Missouri. Environ Sci Technol 50: 5781-5789.

Uren Webster, T.M. & Santos, E.M. (2015). Global transcriptomic profiling demonstrates induction of oxidative stress and of compensatory cellular stress response in brown trout exposed to glyphosate and Roundup. BMC Genomics 16: 32.

USDA (2007). Draft Environmental Assessment. In response to Pioneer Hi-Bred International petition 06-271-01p seeking a determination of nonregulated status for herbicide tolerant 356043 soybean. http://www.aphis.usda.gov/brs/aphisdocs/06_27101p_pea.pdf

USDA (2014). Dow AgroSciences Petitions (09-233-01p, 09-349-01p, and 11-234-01p) for determinations of nonregulated Status for 2,4-D-resistant corn and soybean varieties, Draft Environmental Impact Statement—2013. http://www.aphis.usda.gov/brs/aphisdocs/24d_deis.pdf

USDA (2015). Determinations of non-regulated status. http://www.aphis.usda.gov/biotechnology/petitions_table_pending.shtml

Vanbergen A. J., The Insect Pollinators Initiative (2013). Threats to an ecosystem service: pressures on pollinators. Frontiers in Ecology and the Environment 11: 251–259.

VanGessel M.J. (2001). Glyphosate-resistant horseweed from Delaware. Weed Science 49: 703-705.

Vencill, W.K., Nichols, R.L., Webster, T.M., Soteres, J.K., Mallory-Smith, C., Burgos, N.R., Johnson, W.G. & McClelland, M.R. (2012). Herbicide resistance: Toward an understanding of resistance development and the impact of herbicide-resistant crops. Weed Science 60, Special Issue: 2-30.

Vera, M.S., Lagomarsino, L., Sylvester, M., Pérez, G.L., Rodríguez, P., Mugni, H., Sinistro, R., Ferraro, M., Bonetto, C., Zagarese, H. & Pizarro, H. (2010). New evidences of Roundup (glyphosate formulation) impact on the periphyton community and the water quality of freshwater ecosystems. Ecotoxicology 19: 710-721.

Verrell, P. & Van Buskirk, E. (2004). As the worm turns: Eisenia foetida avoids soil contaminated by a glyphosate-based herbicide. Bull. Environ. Contam. Toxicol. 72: 219-224.

Vila-Aiub, M.M., Vidal, R.A., Balbi, M.C., Gundel, P.E., Trucco, F. & Ghersa, C.M. (2008). Glyphosate-resistant weeds of South American cropping systems: an overview. Pest Manag Sci. 64: 366-371.

Vitta, J.I., Tuesca, D. & Puricelli, E. (2004). Widespread use of glyphosate tolerant soybean and weed community richness in Argentina. Agricult. Ecosyst. Environm. 103: 621-624.

Wade, L. (2104). Monarch numbers in Mexico fall to record low. <http://news.sciencemag.org/biology/2014/01/monarch-numbers-mexico-fall-record-low>

Waggoner, B.S., Mueller, T.C., Bond, J.A. & Steckel, L.E. (2011). Control of glyphosate-resistant horseweed (Conyza Canadensis) with saflufenacil tank mixtures in no-till cotton. Weed Technology 25: 310-315.

Wagner, N. & Lötters, S. (2013). Possible correlation of the worldwide amphibian decline and the increasing use of glyphosate in the agrarian industry. BfN-Skripten 343. http://bfn.de/0502_skripten.html.

Wagner, N., Reichenbecher, W., Teichmann, H., Tappeser, B. & Lötters, S. (2013). Questions concerning the potential impact of glyphosate-based herbicides on amphibians. Env. Toxicol. Chemistry 32: 1688–1700.

Wang, W., Xia, H., Yang. X, Xu, T., Si, H.J., Cai, X.X., Wang, F., Su, J., Snow, A.A., Lu, B.-R. (2014). A novel 5-enolpyruvoylshikimate-3-phosphate (EPSP) synthase transgene for glyphosate resistance stimulates growth and fecundity in weedy rice (Oryza sativa) without herbicide. New Phytologist 202: 679–683.

Wardle, D. A., Nicholson, K. S., Bonner, K. I. & Yeates, G.W. (1999). Effects of agricultural intensification on soil-associated arthropod population dynamics, community structure, diversity and temporal variability over a seven-year period. Soil Biol Biochem 31: 1691-1706.

Watkinson, A.R., Freckleton, R.P., Robinson, R.A. & Sutherland, W.J. (2000). Predictions of Biodiversity Response to Genetically Modified Herbicide-Tolerant Crops. Science 289: 1554-1557.

Webster, T.M. & Nichols, R.L. (2012). Changes in the prevalence of weed species in the major agronomic crops of the Southern United States: 1994/1995 to 2008/2009. Weed Science 60: 145-157.

Werner, B. & Garbe, V. (1998). Bedeutung der Unkrautverteilung im Winterraps für eine gezielte Bekämpfung nach Schadensschwellen, Z. PflKrankh. PflSchutz, Sonderheft XVI: 279-288.

WHO (1994). Environmental health criteria 159: Glyphosate. International Programme of Chemical Safety. http://www.inchem.org/documents/ehc/ehc/ehc159.htm.

WHO (2005). Glyphosate and AMPA in drinking water. <http://www.who.int/water_sanitation_health/dwq/chemicals/glyphosateampa290605.pdf>

Wilson, R.S., Tucker, M.A., Hooker, N.H., LeJeune, J.T. & Doohan, D. (2008). Perceptions and belief about weed management: perspectives of Ohio grain and produce farmers. Weed Technol 22: 339-350.

WSSA (Weed Science Society of America). (1998). Herbicide Resistance and Herbicide Tolerance Definitions. Weed Technol 12 (4): 789. http://wssa.net/wssa/weed/resistance/herbicide-resistance-and-herbicide-tolerance-definitions/olfe, M.S. (2000). Crop strength through diversity. Nature 406: 681-682.

Wright, T.R., Shan, G., Walsh, T.A., Lira, J.M., Cui, C., Song, P., Zhuang, M., Arnold, N.L., Lin, G., Yau, K. et al. (2010). Robust crop resistance to broadleaf and grass herbicides provided by aryloxyalkanoate dioxygenase transgenes. PNAS 107: 20240-20245.

Yuan, J.S., Tranel, P.J. & Stewart Jr., C.N. (2007). Non-target-site herbicide resistance: a family business. Trends in Plant Science 12: 6-13.

Zablotowicz, R.M. & Reddy, K.N. (2007). Nitrogenase activity, nitrogen content, and yield responses to glyphosate in glyphosate-resistant soybean. Crop Protection 26: 370-376.

Zelaya, I.A., Owen, M.D.K. & VanGessel, M.J. (2007). Transfer of glyphosate resistance: evidence of hybridization in Conyza (Asteraceae). American Journal of Botany 94: 660-673.

Zentner, R.P., Wall, D.D., Nagy, C.N., Smith, E.G., Young, D.L., Miller, P.R., Campell, C.A., McConkey, B. G., Brandt, S.A., Lanfond, G.P, et al. (2002). Economics of crop diversification and soil tillage opportunities in the Canadian prairies. Agron J 94: 216-230.

Zhou, X.V., Larson, J.A., Lambert, D.M., Roberts, R.K. & English, B.C. (2015). Farmer experience with weed resistance to herbicides in cotton production. AgBioForum 18(1): 114-125. <https://mospace.umsystem.edu/xmlui/handle/10355/45533>

Zobiole, L.H.S., Kremer, R.J., Oliveira, R.S. & Constantin, J. (2010). Glyphosate affects photosynthesis in first and second generation of glyphosate-resistant soybeans. Plant Soil 336: 251-265.

Zobiole, L.H. S.; Kremer, R.J.; Oliveira, R.S., Jr. & Constantin, J. (2011a). Glyphosate affects micro-organisms in rhizospheres of glyphosate-resistant soybean. Journal of Applied Microbiology, 110: 118-127.

Zobiole, L.H.S., Kremer, R.J., Oliveira, R.S. & Constantin, J. (2011b). Glyphosate affects chlorophyll, nodulation and nutrient accumulation of “second generation” glyphosate-resistant soybean (Glycine max L). Pesticide Biochemistry and Physiology 99: 53-60.

Zobiole, L. H. S., Kremer, R.J., Oliveira Jr., R. S. & Constantin, J. (2012) Glyphosate effects on photosynthesis, nutrient accumulation, and nodulation in glyphosate-resistant soybean. Journal of Plant Nutrition and Soil Science, 175: 319–330.

1. The European Networks of the Heads of Environment Protection Agencies EPA and European Nature Conservation Agencies ENCA. The subset of the Interest Group GMO consisted of the Environment Agency Austria EAA, the Finnish Environment Institute SYKE, the German Federal Agency for Nature Conservation BfN, the Institute for Environmental Protection and Research ISPRA, and the Swiss Federal Office for the Environment FOEN. [↑](#footnote-ref-2)
2. Insert link [↑](#footnote-ref-3)
3. http://www.cbd.int [↑](#footnote-ref-4)
4. The CPB uses LMO instead of GMO, restricting the scope to only living modified organisms [↑](#footnote-ref-5)
5. Throughout this document the terms "herbicide resistance" and "herbicide tolerance" are used as defined by the Weed Science Society of America (WSSA 1998); both terms are not used synonymously with respect to a particular response to a herbicide, they rather distinguish naturally occurring "tolerance" from engineered "resistance". [↑](#footnote-ref-6)
6. e.g. maize Bt11xGA21, MON 89034×1507×NK603, cotton GHB614xLL25xMON15985 [↑](#footnote-ref-7)
7. 2,4-dichlorphenoxyacetic acid [↑](#footnote-ref-8)
8. hydroxyphenylpyruvate dioxygenase [↑](#footnote-ref-9)
9. acetolactate synthase (ALS) [↑](#footnote-ref-10)
10. e.g. soybean DAS-44406-6, cotton DAS-81910-7 [↑](#footnote-ref-11)
11. Inhibitor of acetyl CoA carboxylase (ACCase) [↑](#footnote-ref-12)
12. <http://www.gmo-compass.org/eng/agri_biotechnology/gmo_planting/344.genetically_modified_rapeseed_global_area_under_cultivation.html> [↑](#footnote-ref-13)
13. First generation RR soybean is RR 40-3-2 and second generation RR soybean is MON 89788 [↑](#footnote-ref-14)
14. http://www.sciencedirect.com/science/journal/02786915/open-access [↑](#footnote-ref-15)
15. http://www.panna.org/sites/default/files/2015-11-24%20EPA%20Voluntary%20Vacatur.pdf, http://www.reuters.com/article/us-agriculture-dow-enlist-idUSKBN0TE25420151125. [↑](#footnote-ref-16)
16. See Figure 20 in Catacora-Vargas et al. 2012 [↑](#footnote-ref-17)
17. <http://stratusresearch.com/blog/glyphosate-resistant-weeds-intensifying> [↑](#footnote-ref-18)
18. <http://sustainablepulse.com/2014/08/21/glyphosate-sales-boom-powers-global-biotech-industry/#.VYF1WPntlBf> [↑](#footnote-ref-19)
19. <http://www.agriculture.com/crops/soybeans/technology/whats-coming-in-herbicidetolert-trait_143-ar43556> [↑](#footnote-ref-20)
20. <http://ec.europa.eu/food/plant/protection/evaluation/existactive/oj_atrazine.pdf> [↑](#footnote-ref-21)
21. Multifunctionality as defined in IAASTD (2008) [↑](#footnote-ref-22)
